# The *Arabidopsis* acetylated histone-binding protein BRAT1 forms a complex with BRP1 and prevents transcriptional silencing

**DOI:** 10.1038/ncomms11715

**Published:** 2016-06-07

**Authors:** Cui-Jun Zhang, Xiao-Mei Hou, Lian-Mei Tan, Chang-Rong Shao, Huan-Wei Huang, Yong-Qiang Li, Lin Li, Tao Cai, She Chen, Xin-Jian He

**Affiliations:** 1 National Institute of Biological Sciences, No. 7, Science Park Road, Zhongguancun Life Science Park, Beijing 102206, China

## Abstract

Transposable elements and other repetitive DNA sequences are usually subject to DNA methylation and transcriptional silencing. However, anti-silencing mechanisms that promote transcription in these regions are not well understood. Here, we describe an anti-silencing factor, Bromodomain and ATPase domain-containing protein 1 (BRAT1), which we identified by a genetic screen in *Arabidopsis thaliana*. BRAT1 interacts with an ATPase domain-containing protein, BRP1 (BRAT1 Partner 1), and both prevent transcriptional silencing at methylated genomic regions. Although BRAT1 mediates DNA demethylation at a small set of loci targeted by the 5-methylcytosine DNA glycosylase ROS1, the involvement of BRAT1 in anti-silencing is largely independent of DNA demethylation. We also demonstrate that the bromodomain of BRAT1 binds to acetylated histone, which may facilitate the prevention of transcriptional silencing. Thus, BRAT1 represents a potential link between histone acetylation and transcriptional anti-silencing at methylated genomic regions, which may be conserved in eukaryotes.

## Abstract (web summary)

Transposons and repetitive sequences are typically subject to transcription silencing. Here, Zhang *et al*. find that the bromodomain-containing protein BRAT1 forms a complex with BRP1, recognizes histone acetylation and acts to prevent transcriptional silencing in *Arabidopsis*.

## Graphical abstract

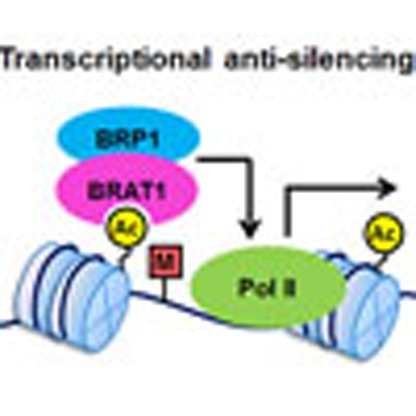

DNA methylation is an important chromatin modification that is required for transposable element (TE) and transgene silencing, genome stability, genomic imprinting and gene regulation^1^,^2^. In *Arabidopsis thaliana*, DNA methylation is enriched on TEs and other repetitive DNA sequences^2^. DNA methylation at symmetric CG sites is catalysed by the maintenance DNA methyltransferase MET1 during DNA replication^3^,^4^. The plant-specific DNA methyltransferase CMT3 is mainly responsible for DNA methylation at CHG (H is A, T, or C) sites^5^,^6^. CMT2 catalyses DDM1-dependent DNA methylation at CHH sites^7^. RNA-directed DNA methylation (RdDM) is responsible for *de novo* DNA methylation^2^,^8^,^9^, in which DNA-dependent RNA polymerase IV and V (Pol IV and Pol V) play critical roles in DNA methylation^10^.

DNA methylation can be either reduced passively during DNA replication or actively by active DNA demethylation^11^,^12^,^13^,^14^. A base excision repair process is involved in active DNA demethylation in plants^11^,^12^,^14^,^15^,^16^. The 5-methylcytosine DNA glycosylase ROS1 is responsible for active DNA demethylation^13^,^17^,^18^,^19^. ROS1 functions redundantly with its homologues DML2 and DML3 in DNA demethylation and thus prevents transcriptional silenicng^17^. DME, another ROS1 homologue, is required for DNA demethylation of imprinted genes in the endosperm^2^,^12^. SSRP1, an HMG domain-containing component of the FACT histone chaperone, is required for active DNA demethylation by DME and contributes to gene imprinting^20^. ROS3, IDM1/ROS4, IDM2/ROS5, IDM3 and MBD7 have been thought to mediate DNA demethylation and repression of transcriptional gene silencing in a ROS1-dependent manner^18^,^21^,^22^,^23^,^24^,^25^,^26^,^27^. MBD7 preferentially binds to highly methylated CG-dense regions and recruits the IDM proteins to methylated genomic regions^21^,^24^,^27^. IDM1 is a histone acetyltransferase, which creates acetylated histone marks required for active DNA demethylation by ROS1 (refs 18, 22). However, it is not known how acetylated histone marks are recognized to mediate DNA demethylation and anti-silencing.

Bromodomain is an acetylated histone interaction module found in various types of nuclear proteins including histone acetyltransferases, transcriptional coactivators and chromatin-remodelling factors^28^,^29^. Twenty-nine bromodomain-containing proteins have been found in *Arabidopsis*^29^. GCN5/HAG1, a histone acetyltransferase, contains a bromodomain that is required for the binding of GCN5 to a subset of its target chromatin loci^30^. The bromodomain protein BRAHMA (BRM) is an SNF2 chromatin-remodelling ATPase that interacts with SWI3C and forms an ATP-dependent chromatin-remodelling complex involved in development, phytohormone signalling and stress response^31^,^32^,^33^. Members of an important subgroup of bromodomain proteins (BET) contain an extra-terminal domain and act as general transcription factors in *Arabidopsis*^29^. Three of these transcription factors, GTE1/IMB1, GTE4 and GTE6, have been functionally characterized and are involved in seed germination, cell division and leaf development, respectively^34^,^35^,^36^. In mammals, BRD4, a BET family member, interacts with acetylated histone and acts as a transcriptional coactivator^37^. The yeast bromodomain protein BDF2 binds to acetylated histone H4 at heterochromatin boundaries and is required to prevent heterochromatin spreading^38^.

The yeast YTA7 protein contains an ATPase domain and a bromodomain^39^. YTA7 binds to the N-terminal tail of histone H3 and is involved in preventing heterochromatin silencing^39^,^40^. LEX-1, a homologue of YTA7 in *C. elegans*, is required for the expression of repetitive genomic sequences, which are usually subjected to transcriptional silencing at heterochromatin regions^41^. The YTA7 homologue in humans, ATAD2/ANCCA, directly interacts with the E2F transcription factors and is required for cell cycle gene expression and cancer cell proliferation^42^,^43^,^44^. Although these results demonstrate that the YTA7 and its homologues are conserved anti-silencing factors in *S. cerevisiae*, *C. elegans* and humans, the function of YTA7 homologues in plants remains to be studied.

In this study, we identify an *Arabidopsis* YTA7 homologue, BRAT1 (Bromodomain and ATPase domain-containing protein 1) as a new anti-silencing factor. We demonstrate that the bromodomain of BRAT1 can bind to acetylated histone and influences histone acetylation levels at methylated genomic regions, thereby providing a potential link between histone acetylation and anti-silencing. Moreover, we demonstrate that BRAT1 associates with a previously uncharacterized ATPase domain-containing protein, BRP1 (BRAT1 Partner 1), and both are required to prevent transcriptional silencing.

## Results

### Identification of BRAT1 as an anti-silencing factor

**Figure 1:**
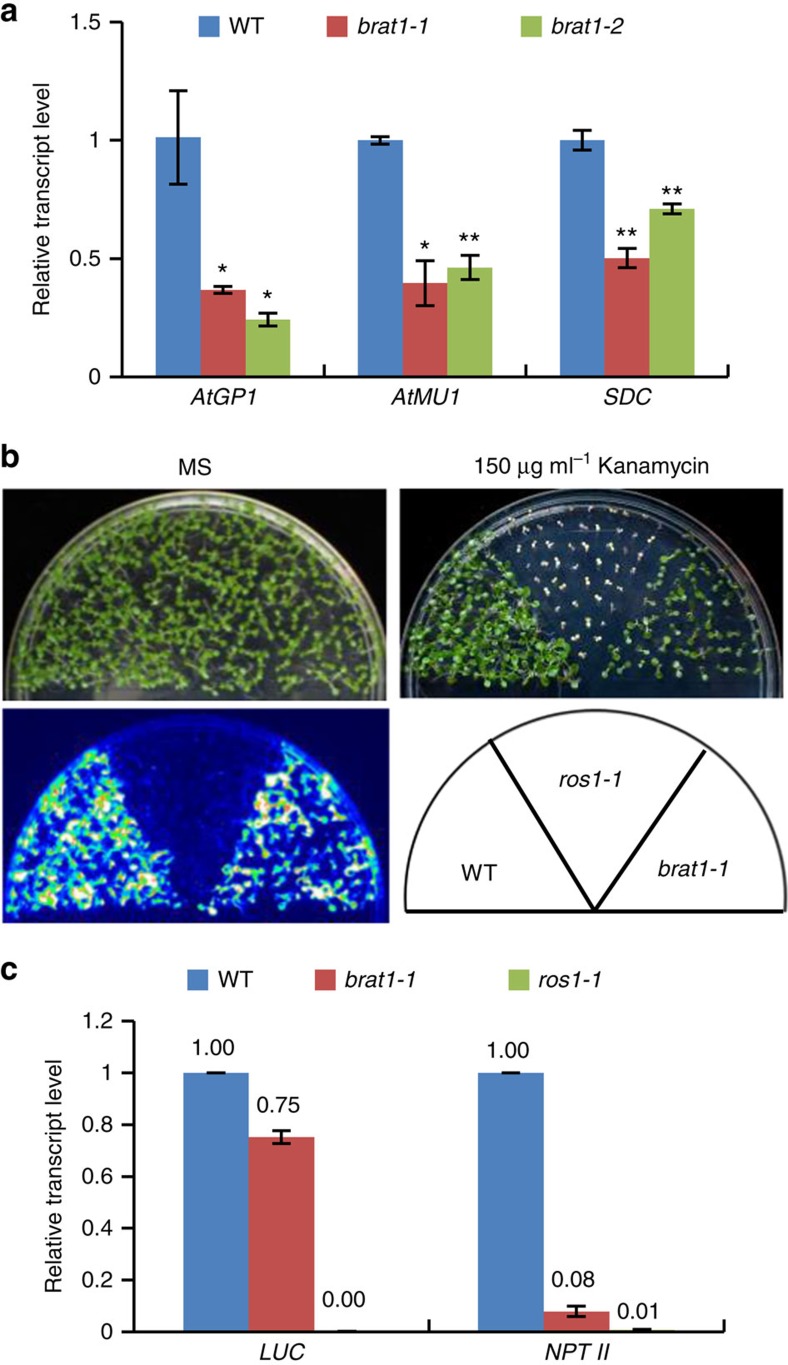
BRAT1 contributes to transcriptional anti-silencing. (**a**) The RNA transcripts of *AtGP1*, *AtMU1* and *SDC* were detected by quantitative RT–PCR in the WT (wild-type) col-0 and the two individual *brat1* mutant alleles *brat1-1* and *brat1-2*. The *actin* gene *ACT7* was amplified as an internal control. Asterisks indicate statistical significance as determined by Student's *t*-test (**P<0.01; *P<0.05). (**b**) The effect of *brat1* on the expression of *RD29A-LUC* and *35S-NPTII* transgenes was determined by luminescence imaging and kanamycin sensitivity, respectively. The *RD29A-LUC* and *35S-NPTII* reporter genes were introduced into the *brat1-1* mutant by crossing. (**c**) The RNA transcripts of *RD29A-LUC* and *35S-NPTII* were detected by quantitative RT–PCR. Error bars represent s.d. of three biological replicates.

*AtGP1* is a gypsy-like retrotransposon that is hypermethylated but not completely silenced in *Arabidopsis*. To identify new regulators in anti-silencing, we screened a collection of homozygous T-DNA insertion mutants by reverse transcription–PCR (RT–PCR) and searched for mutants in which the transcript level of *AtGP1* is decreased. The collection included mutants of 550 chromatin-related genes (Supplementary Data 1). We identified a mutant (Salk_012966C, *brat1-1*) with a T-DNA insertion in the gene *AT1G05910* (Supplementary Fig. 1a–c). We refer to this gene as *BRAT1* (*Bromodomain-containing protein with ATPase domain 1*). We tested the transcript level of *AtGP1* in another *brat1* T-DNA mutant allele (Salk_134173C, *brat1-2*; Supplementary Fig. 1a–c) and found that the transcript level of *AtGP1* is also markedly decreased in *brat1-2* compared with wild type (Fig. 1a and Supplementary Fig. 1d). We transformed *BRAT1-Flag* into the *brat1-1* mutant, and found that the transcript level of *AtGP1* is completely restored by *BRAT1-Flag* in the *brat1* mutant (Supplementary Fig. 1e). In addition, we found that the transcript levels of the retrotransposon *AtMU1* and the DNA repeat-flanking gene *SDC* are reduced in the *brat1-1* and *brat1-2* mutants (Fig. 1a). These results suggest that BRAT1 is an anti-silencing factor that prevents silencing of the *AtGP1*, *AtMU1* and *SDC* loci.

Previous studies have shown that the 5-methylcytosine DNA glycosylase ROS1 is required to prevent the silencing of both *RD29A-LUC* and *35S-NPTII* transgenes^11^,^13^,^14^, whereas the histone H3 acetyltransferase IDM1 and the α-crystallin domain protein IDM2 are required to prevent the silencing of *35S-NPTII* but not *RD29A-LUC*^18^,^22^,^23^,^25^. To determine whether BRAT1 functions in anti-silencing of transgenes, we introduced the *brat1-1* mutation into the *RD29A-LUC* and *35S-NPTII* transgenic plants by crossing. The results indicated that the expression of *35S-NPTII* is markedly repressed in the *brat1-1* mutant, resulting in decreased kanamycin resistance of the mutant relative to the wild type (Fig. 1b,c). The expression of *RD29A-LUC* is only slightly affected in the *brat1-1* mutant (Fig. 1b,c). The result suggests that the *brat1* mutation preferentially affects *35S-NPTII* rather than *RD29A-LUC*. Thus, the function of BRAT1 in anti-silencing of transgenes is comparable to that of IDM1 and IDM2.

### BRAT1 and BRP1 form a complex

**Figure 2:**
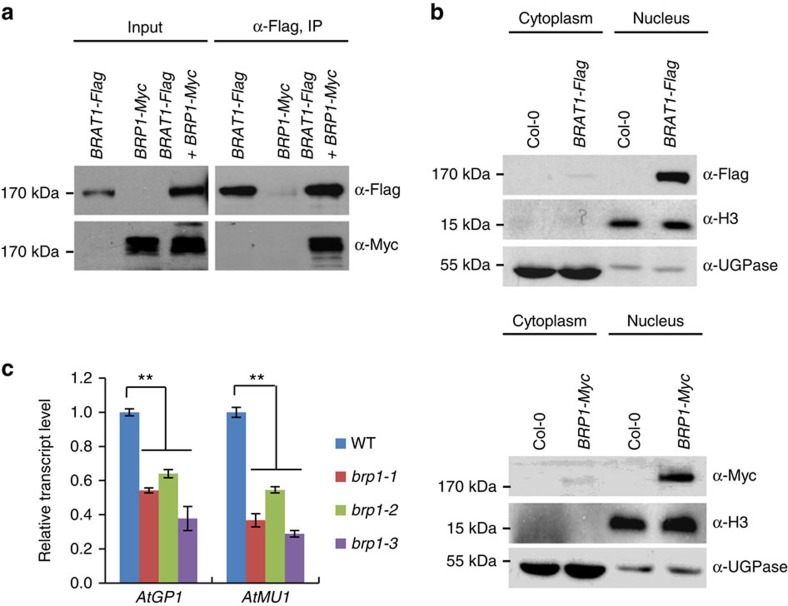
BRAT1 associates with BRP1 and both are involved in transcriptional anti-silencing. (**a**) The interaction between BRAT1-Flag and BRP1-Myc as determined by co-IP. *BRAT1-Flag* transgenic plants were crossed to *BRP1-Myc* transgenic plants, and the F2 lines expressing both fusion proteins were subjected to co-IP. (**b**) The cytoplasmic and nuclear fractions of wild-type and transgenic seedlings were analysed by western blotting with histone H3 (nuclear marker), UGPase (cytoplasmic marker), Flag and Myc antibodies. Protein size markers are indicated in kDa. (**c**) The RNA transcripts of *AtGP1* and *AtMU1* were detected by quantitative RT–PCR in the wild type and three individual *brp1* mutant alleles. Asterisks indicate statistical significance as determined by Student's *t*-test (**P<0.01). *ACT7* was amplified as an internal control. Error bars represent s.d. of three biological replicates.

To investigate BRAT1 function, we performed affinity purification to isolate proteins by anti-Flag antibody from the *BRAT1-Flag* transgenic plants. By mass spectrometric analysis, we found that BRAT1 co-purified a previously uncharacterized protein (AT3G15120), which we refer to as BRAT1 Partner 1 (BRP1; Table 1). We generated *BRP1-Myc* transgenic plants and purified proteins associated with BRP1 by affinity purification. Mass spectrometric analysis indicated that BRP1 also co-purified BRAT1 (Table 1). We crossed *BRP1-Myc* transgenic plants to *BRAT1-Flag* transgenic plants to obtain F1 plants harbouring both transgenes for co-immunoprecipitation (co-IP). The result indicated that BRP1-Myc and BRAT1-Flag were co-precipitated by anti-Flag antibody (Fig. 2a), confirming that BRAT1 can interact with BRP1 *in vivo*. We further produced transgenic plants harbouring both *BRAT1-Flag* and *ROS1-Myc* transgenes for co-IP. An interaction between BRAT1 and ROS1 was not detected (Supplementary Fig. 2). We extracted cytoplasmic and nuclear fractions to determine the subcellular localization of BRAT1 and BRP1 and found that both proteins were predominantly detected in the nucleus (Fig. 2b). In addition, we extracted nucleoplasmic and chromatin fractions to determine the subnuclear localization of BRAT1 and BRP1. Western blot analysis shows that BRAT1 and BRP1 both exist in the chromatin fraction but not in the nucleoplasmic fraction (Supplementary Fig. 3), suggesting that the BRAT1–BRP1 complex associates with chromatin.

BRP1 contains a plant homeodomain-like zinc finger domain and an AAA-type ATPase domain (Supplementary Fig. 4a). Plant homeodomains usually act as modules for interacting with chromatin or chromatin-related proteins, whereas the AAA-type ATPase domain is conserved in BRP1 and BRAT1. We investigated whether BRP1 has the same biological function as BRAT1 in anti-silencing. Quantitative RT–PCR demonstrated that the transcript levels of *AtGP1* and *AtMU1* are significantly reduced in three individual *brp1* mutant alleles including *brp1-1* (Salk_021560C), *brp1-2* (Salk_059263C) and *brp1-3* (Salk_046896C; Fig. 2c; Supplementary Fig. 4b–d). These results suggest that both BRP1 and BRAT1 function in anti-silencing.

### BRAT1 and BRP1 contribute to DNA demethylation at some loci

**Figure 3:**
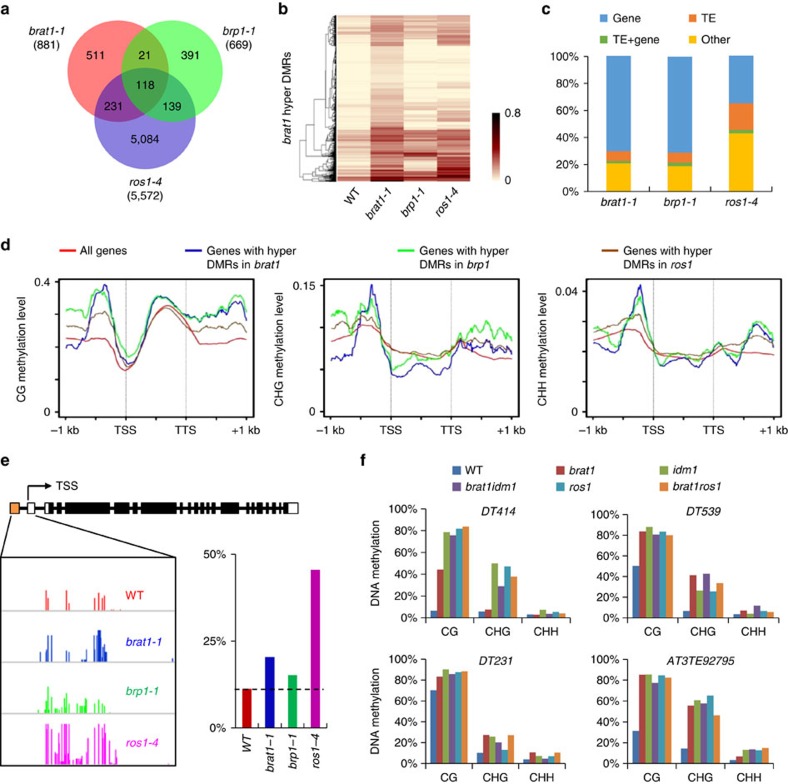
DNA demethylation loci targeted by BRAT1 and BRP1. (**a**) Numbers of overlapping hyper-DMRs among *brat1-1*, *brp1-1* and *ros1-4* mutants. (**b**) Heat maps of DNA methylation levels within *brat1-1* hyper-DMRs. The columns represent the indicated genotypes, and the rows represent the differentially methylated loci. Light yellow indicates low methylation, and black indicates high methylation. (**c**) Composition of the genomic location of the hyper-DMRs in the *brat1-1*, *brp1-1* and *ros1-4* mutants. TE, transposable element. (**d**) Plots indicate CG, CHG and CHH methylation at genes and their 1-kb upstream and downstream regions in the wild type (WT). Red lines indicate DNA methylation of all genes in *Arabidopsis*, and blue, green and brown lines indicate DNA methylation of genes with hyper-DMRs in *brat1*, *brp1* and *ros1*, respectively. TSS, transcription start site; TTS, transcription termination site. (**e**) The DNA methylation level of the *ROS1* promoter region was calculated based on the whole-genome bisulfite sequencing data. The methylation levels of the boxed region are shown by the histograms for WT, *brat1-1*, *brp1-1* and *ros1-4*. The yellow box indicates the TE region in the *ROS1* promoter. (**f**) The DNA methylation levels of *DT414*, *DT231*, *DT539* and *AT3TE92795* as determined by locus-specific bisulfite sequencing analyses.

The 5-methylcytosine DNA glycosylase ROS1 and its close homologues, DML2 and DML3, function in active DNA demethylation^17^. In the DNA demethylation mutants *ros1dml2dml3* (*rdd*), *ros1* and *idm1*, thousands of DNA hypermethylated loci were previously identified by genome-wide DNA methylation analyses^4^,^17^,^18^. We performed whole-genome DNA methylation analyses by bisulfite sequencing and obtained 2.9 × 10^7^, 3.1 × 10^7^, 4.9 × 10^7^ and 4.6 × 10^7^ reads for the wild type, *brat1-1*, *brp1-1* and *ros1-4*, respectively (Supplementary Table 1). Whole-genome DNA methylation analyses identified 5,572, 881 and 669 hypermethylated differentially methylated regions (hyper-DMRs) in *ros1*, *brat1-1* and *brp1-1*, respectively (Fig. 3a and Supplementary Data 2). The numbers of hyper-DMRs are much less in *brat1* and *brp1* than in *ros1* (Fig. 3a), suggesting that BRAT1 and BRP1 contribute to DNA demethylation at a smaller set of loci than targeted by ROS1. Venn diagrams indicate that 39.6% (349/881) and 38.4% (257/669) of the hyper-DMRs in *brat1* and *brp1*, respectively, overlap with the hyper-DMRs in *ros1* (Fig. 3a), which are significantly higher than expected by chance (6/881=0.68% in *brat1* and 5/669=0.75% in *brp1*; *P*<0.01; hypergeometric test). Heat maps indicate that hyper-DMRs in *brat1* are often preferentially hypermethylated in *ros1* and *brp1* (Fig. 3b). For the *brat1*- and *brp1*-specific hyper-DMRs (Fig. 3a), their average DNA methylation levels are also weakly increased in *ros1* (Supplementary Fig. 5). Because ROS1 has homologues, it is possible that the BRAT1 and BRP1 target loci not significantly regulated by ROS1 may be regulated by ROS1 homologues.

*AT1G26400* and *AT4G18650* are among hyper-DMRs identified in *rdd*, *ros1* and *idm1* in a previous report^18^. Using PCR-based DNA methylation analyses, we found that their DNA methylation levels are increased in two independent *brat1* mutant alleles as well as in *ros1* (Supplementary Fig. 6a). The DNA methylation levels were reduced by the *BRAT1-Flag* transgene in the *brat1* mutant background (Supplementary Fig. 6b). Moreover, we found that DNA methylation of *AT4G18650* is markedly increased in three independent *brp1* mutant alleles as well as in the *brat1* mutant (Supplementary Fig. 6c). The *BRP1-Myc* transgene complements the DNA hypermethylation phenotype of the *brp1* mutant (Supplementary Fig. 6d). These results suggest that BRAT1 and BRP1 are related to DNA demethylation at these loci.

Previous reports demonstrated that a large number of hypermethylated loci in *ros1* and/or *rdd* are close to or overlap with genes^17^,^18^,^45^. Our bisulfite sequencing data indicate that more than 70% (621/881 for *brat1* and 476/669 for *brp1*) of hyper-DMRs in *brat1* and *brp1* overlap with genic regions, whereas 35.3% (1,966/5,572) of hyper-DMRs in *ros1* overlap with genic regions (Fig. 3c). The DNA demethylation target genes show much higher DNA methylation levels than other genes at 5′-promoter and 3′-terminator regions for all three cytosine contexts (Fig. 3d). It is possible that these hypermethylated genes require DNA demethylation and anti-silencing mechanisms to prevent transcriptional silencing.

Two recent studies reported that DNA methylation of a regulatory element in the *ROS1* promoter region is dynamically regulated by RdDM and DNA demethylation and plays a positive role in *ROS1* expression^46^,^47^. In the *ros1* mutant, DNA methylation of the *ROS1* promoter region is upregulated and then promotes the expression of *ROS1*. Our whole-genome DNA methylation data show that DNA methylation of the *ROS1* promoter region is upregulated not only in *ros1* but also in *brat1* and *brp1* (Fig. 3e), which is consistent with the result showing that *ROS1* expression is induced in *ros1*, *brat1* and *brp1* (Supplementary Fig. 7). These results suggest that BRAT1 and BRP1, in addition to ROS1, contribute to DNA demethylation of the *ROS1* promoter region and thereby may contribute to transcriptional repression of *ROS1*.

To examine the genetic relationship between *brat1*, *idm1* and *ros1*, we crossed *brat1* to *idm1* and *ros1*, thus obtaining the double mutants *brat1idm1* and *brat1ros1*. *DT414*, *DT231*, *DT539* and *AT3TE92795* are hypermethylated loci in *brat1*, *idm1* and *ros1* as determined by the whole-genome bisulfite sequencing data (Supplementary Data 2). Our locus-specific bisulfite sequencing analysis further confirmed that the DNA methylation levels of these loci are markedly increased in *brat1*, *idm1* and *ros1* relative to the wild type (Fig. 3f). However, the DNA methylation levels are not further enhanced in the double mutants *brat1idm1* and *brat1ros1* relative to each of the single mutants (Fig. 3f). The results suggest that BRAT1 may contribute to DNA demethylation through the same genetic pathway as IDM1 and ROS1.

### DNA demethylation is mostly dispensable for anti-silencing

**Figure 4:**
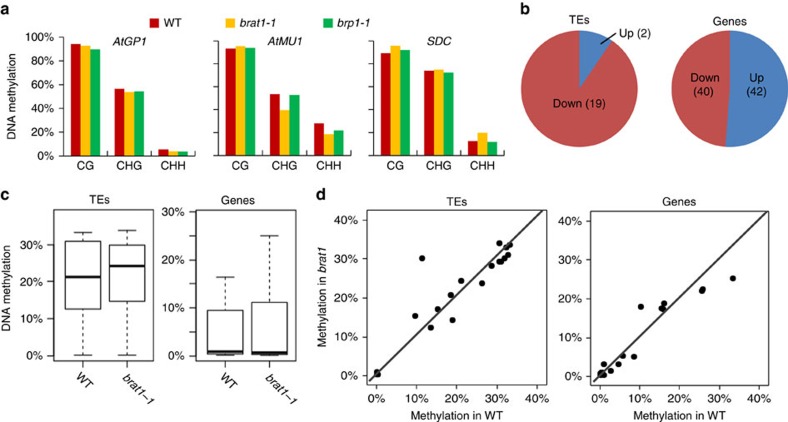
BRAT1 and BRP1 affect the transcript levels of their target loci without alteration of DNA methylation. (**a**) The DNA methylation levels of *AtGP1*, *AtMU1* and *SDC* were calculated from the whole-genome bisulfite sequencing data. The percentages of CG, CHG and CHH methylation are separately shown. (**b**) Numbers of TEs and protein-coding genes up- or downregulated in *brat1* relative to the wild type (WT) as determined by the RNA deep sequencing analysis. (**c**,**d**) DNA methylation levels of downregulated TEs and genes in *brat1* were indicated by box plots and scatter plots.

The results shown above demonstrated that BRAT1 functions in transcriptional anti-silencing of *AtGP1*, *AtMU1* and *SDC* (Fig. 1a). Given that BRAT1 appears to contribute to DNA demethylation at some loci, many of which are also targeted by *ROS1*, we initially expected that DNA demethylation would be required for the function of BRAT1 in anti-silencing. However, our whole-genome DNA methylation analysis indicated that these three loci are highly methylated in the wild type and their DNA methylation levels are not enhanced in *brat1-1*, *brp1-1* and *ros1-4* in all three cytosine contexts CG, CHG and CHH (Fig. 4a). These results suggest that BRAT1 affects the transcript levels of *AtGP1*, *AtMU1* and *SDC* in a DNA demethylation-independent manner.

We performed RNA deep sequencing assay with three replicates for each genotype to identify loci affected in the *brat1* mutant (Supplementary Table 2). In total, we identified 21 TEs (*P*<0.05; log2(fold-change) >1 or <−1; Cufflinks) and 82 genes (*P*<0.01; log2(fold-change)>1 or <−1; Cufflinks) that are differently expressed in *brat1* relative to the wild type (Fig. 4b; Supplementary Data 3). Gene Ontology analyses show that stress response genes are enriched in the differentially expressed genes in *brat1* (Supplementary Fig. 8). Among the 21 differentially expressed TEs, the majority (19/21) are decreased (Fig. 4b and Supplementary Data 3), which is consistent with the notion that BRAT1 is involved in transcriptional anti-silencing. Of the differentially expressed genes in *brat1*, 48.8% (40/82) show reduced expression, and the remainder show increased expression (Fig. 4b and Supplementary Data 3).

To determine whether reduced expression of TEs and genes is correlated with DNA hypermethylation in *brat1*, we compared the RNA deep sequencing data with the whole-genome DNA methylation data and found that downregulated TEs and genes do not significantly overlap with hyper-DMRs in *brat1* (Supplementary Data 2 and 3). The DNA methylation levels of most transcriptionally downregulated TEs and genes are not affected in *brat1* relative to the wild type (Fig. 4c,d). The results suggest that the involvement of BRAT1 in anti-silencing is largely independent of DNA demethylation even though BRAT1 contributes to DNA demethylation at a specific subset of loci targeted by ROS1.

DNA demethylation caused by ROS1 and IDM1 was shown to prevent transcriptional silencing^13^,^18^. However, for most of DNA demethylation target loci, their transcript levels are below the detectability of our RNA deep sequencing analysis. We randomly selected seven hyper-DMRs shared in *brat1*, *brp1* and *ros1* to determine whether DNA hypermethylation is correlated with reduced expression (Supplementary Fig. 9). The effect of *brat1*, *brp1* and *ros1* on DNA methylation was confirmed by PCR-based DNA methylation assay (Supplementary Fig. 10a,b). The transcript levels of the seven hyper-DMRs were determined by quantitative RT–PCR. Of the seven hyper-DMRs, the transcript levels of four loci are reduced in *brat1*, *brp1* and *ros1* relative to the wild type, whereas the transcript levels of three others are not reduced (Supplementary Fig. 10c,d). These results suggest that DNA demethylation is correlated with anti-silencing only at a subset of target loci shared by BRAT1, BRP1 and ROS1.

### *brat1* affects transcriptional de-repression in RdDM mutants

**Figure 5:**
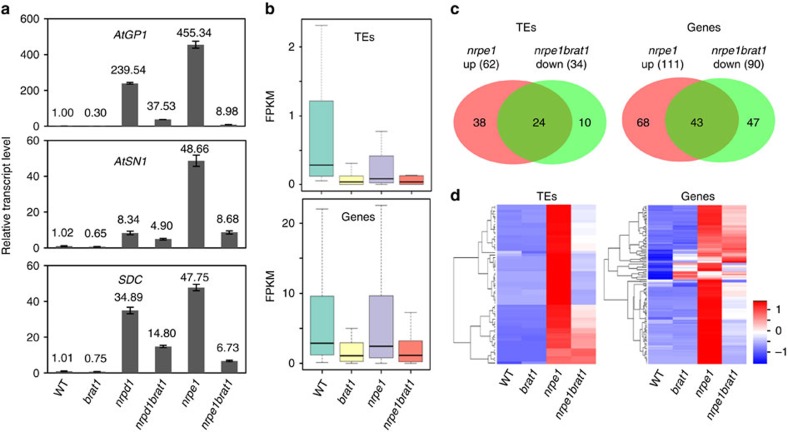
BRAT1 contributes to the expression of RdDM target loci in the RdDM mutants. (**a**) The RNA transcripts of *AtGP1*, *AtSN1* and *SDC* were detected by quantitative RT–PCR in the indicated genotypes. Error bars are s.d. of three technical replicates in a representative experiment. (**b**) Box plots showing the expression of TEs and protein-coding genes that are downregulated in *brat1*. FPKM, fragments per kilo base of exons per million fragments mapped. (**c**) Venn diagrams of overlap between upregulated loci in *nrpe1* versus wild type (WT) and downregulated loci in *brat1nrpe1* versus *nrpe1*. (**d**) Heat maps showing RNA transcripts of upregulated (log_2_(fold-change of reads)>1, *P*<0.01,Cufflinks) TEs and genes in *nrpe1* compared with the WT. The columns represent the indicated genotypes, and the rows represent the relative RNA transcript levels. Blue indicates low expression, and red indicates high expression.

*AtGP1*, *AtSN1* and *SDC* are known RdDM target loci and show increased expression in the RdDM mutants *nrpd1* and *nrpe1* relative to the wild type^48^,^49^,^50^,^51^. Our quantitative RT–PCR experiment demonstrated that the *brat1* mutation moderately reduces the transcript levels of these loci (Figs 1a and 5a). We crossed *brat1-1* to *nrpd1* and *nrpe1* to obtain *brat1nrpd1* and *brat1nrpe1* double mutants. We found that while the transcript levels of *AtGP1*, *AtSN1* and *SDC* are dramatically increased in either the *nrpd1* or the *nrpe1* single mutant, their transcript levels are markedly decreased in either the *brat1nrpd1* or the *brat1nrpe1* double mutant (Fig. 5a). The results suggest that BRAT1 contributes to increased expression of *AtSN1*, *AtGP1* and *SDC* in the *nrpd1* and *nrpe1* background, indicating an anti-silencing function of BRAT1 at these RdDM target loci in these genetic backgrounds.

Our RNA deep sequencing analysis demonstrate that many loci are transcriptionally downregulated in the *brat1* mutant and that the transcript levels of these loci are not induced in the RdDM mutants (Figs 4b and 5b and Supplementary Data 3). By analysing published whole-genome DNA methylation data^4^, we found that on average, DNA methylation levels of loci transcriptionally downregulated in *brat1* are significantly reduced in the canonical RdDM mutant *nrpd1* relative to the wild type (Supplementary Fig. 11 and Supplementary Data 3), suggesting that DNA methylation of loci downregulated in *brat1* may be affected by the RdDM pathway. We therefore predict that DNA methylation of BRAT1 target loci may in some cases be partly regulated by RdDM. However, there are also likely to be other silencing mechanisms as disruption of the RdDM pathway is insufficient for transcriptional derepression of BRAT1 target loci.

**Figure 6:**
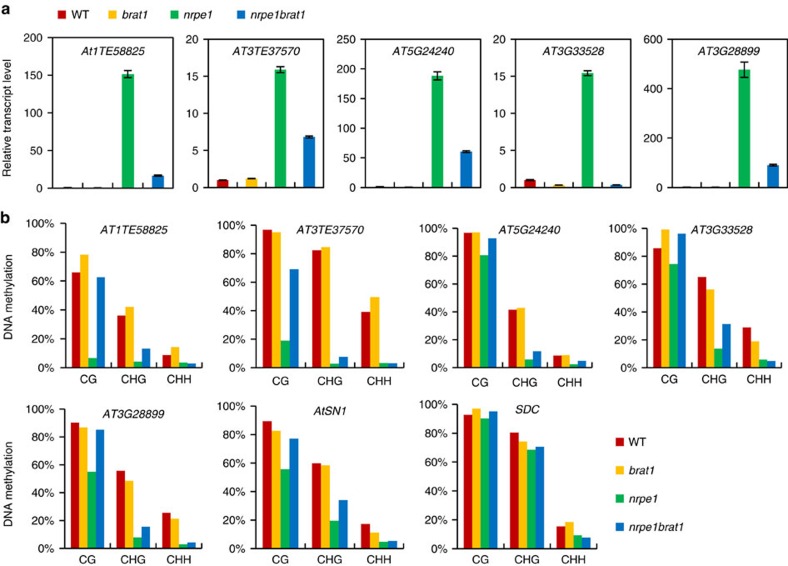
Involvement of BRAT1 in *nrpe1*-induced expression is either dependent or independent of DNA demethylation. (**a**) The RNA transcripts of *AT1TE58825*, *AT3TE37570*, *AT5G24240*, *AT3G33528* and *AT3G28899* were evaluated by quantitative RT–PCR in wild type (WT), *brat1*, *nrpe1* and *nrpe1brat1*. Error bars are s.d. of three technical replicates in a representative experiment. (**b**) DNA methylation was determined by locus-specific bisulfite sequencing at *AtSN1*, *SDC*, *AT1TE58825*, *AT3TE37570*, *AT5G24240*, *AT3G33528* and *AT3G28899*. The percentages of CG, CHG and CHH methylation are separately shown.

As determined by our RNA deep-sequencing analysis, the loci that are downregulated in *brat1* single mutants are, on average, also downregulated in the *brat1nrpe1* double mutant (Fig. 5b), providing further evidence that BRAT1 is required for preventing transcriptional silencing at these loci. Our RNA deep-sequencing analysis identified 62 TEs and 111 genes that are upregulated in the RdDM mutant *nrpe1* (Fig. 5c and Supplementary Data 4). We found that the transcript levels of a large number of the upregulated loci are reduced in the *brat1nrpe1* double mutant (Fig. 5c,d and Supplementary Data 4 and 5), suggesting that BRAT1 contributes to the expression of RdDM target loci in the *nrpe1* mutant background. However, the expression of the RdDM target loci is not significantly affected by *brat1* in the wild-type background. The effect of *brat1* on the expression of RdDM target loci in the *brat1nrpre1* double mutant was confirmed by quantitative RT–PCR at five randomly selected RdDM target loci (Fig. 6a). We performed locus-specific bisulfite sequencing to determine whether the effect of *brat1* on the expression of the five RdDM target loci in the *brat1nrpe1* mutant is correlated with DNA methylation. Our results demonstrated that while the DNA methylation levels of the five RdDM target loci are reduced at all the three cytosine contexts (CG, CHG and CHH) in the *nrpe1* mutant, the reduction of DNA methylation in the five RdDM target loci is restored at CG sites and to a lesser extent at CHG sites in the *brat1nrpe1* double mutant (Fig. 6b). Meanwhile, we found that DNA methylation of the five RdDM target loci is either not induced or only slightly induced in the *brat1* single mutant relative to the wild type (Fig. 6b), which is consistent with the notion that BRAT1 does not contribute to anti-silencing of these RdDM loci via DNA demethylation in the wild-type background.

*AtSN1* and *SDC* are two loci whose transcript levels are reduced by *brat1* not only in the *brat1* single mutant but also in the *brat1nrpe1* double mutant (Fig. 5a). We determined the DNA methylation levels of the two loci by bisulfite sequencing analyses. In the *brat1* single mutant, the DNA methylation levels of *AtSN1* and *SDC* are not significantly changed even though the transcript levels of the two loci are reduced (Figs 5a and 6b). In the *nrpe1* single mutant, DNA methylation of *AtSN1* is markedly reduced at all the cytosine contexts (Fig. 6b). In the *brat1nrpe1* double mutant, the reduction of *AtSN1* methylation caused by *nrpe1* is restored at CG sites and to a lesser extent at CHG sites (Fig. 6b). In the *nrpe1* single mutant, the DNA methylation level of *SDC* is significantly reduced at CHH sites but not at CG and CHG sites (Fig. 6b). In the *brat1nrpe1* double mutant, the DNA methylation level of *SDC* is not affected even though the transcript level of *SDC* is repressed (Fig. 6b). Thus, we suggest that in the *nrpe1* mutant background, the effect of *brat1* on transcript levels of RdDM target loci is correlated with DNA methylation levels only at a subset of RdDM target loci. Taken together, these results suggest that the involvement of BRAT1 in anti-silencing is not primarily related to DNA demethylation and that BRAT1 has a DNA demethylation-independent role in anti-silencing.

To determine whether the *ros1* mutation could reduce the expression of RdDM target loci in the *nrpe1* mutant background, we crossed *ros1-4* to *nrpe1-11* and obtained the double mutant *ros1nrpe1*. Quantitative RT–PCR data show that the transcript levels of some RdDM target loci (*AtSN1*, *AT1TE58825*, *AT3TE37570*, *AT5G24240*, *AT3G33528* and *AT3G28899*) are markedly decreased in the *ros1nrpe1* double mutant (Supplementary Fig. 12a). PCR-based DNA methylation analyses indicate that the reduction of DNA methylation in the *nrpe1* mutant is partially restored by *ros1* in the *ros1nrpe1* double mutant (Supplementary Fig. 12b). This results suggest that, like BRAT1, ROS1 contributes to the expression of some RdDM target loci in the *nrpre1* mutant background.

### The bromodomain of BRAT1 binds to the acetylated histone H4

**Figure 7:**
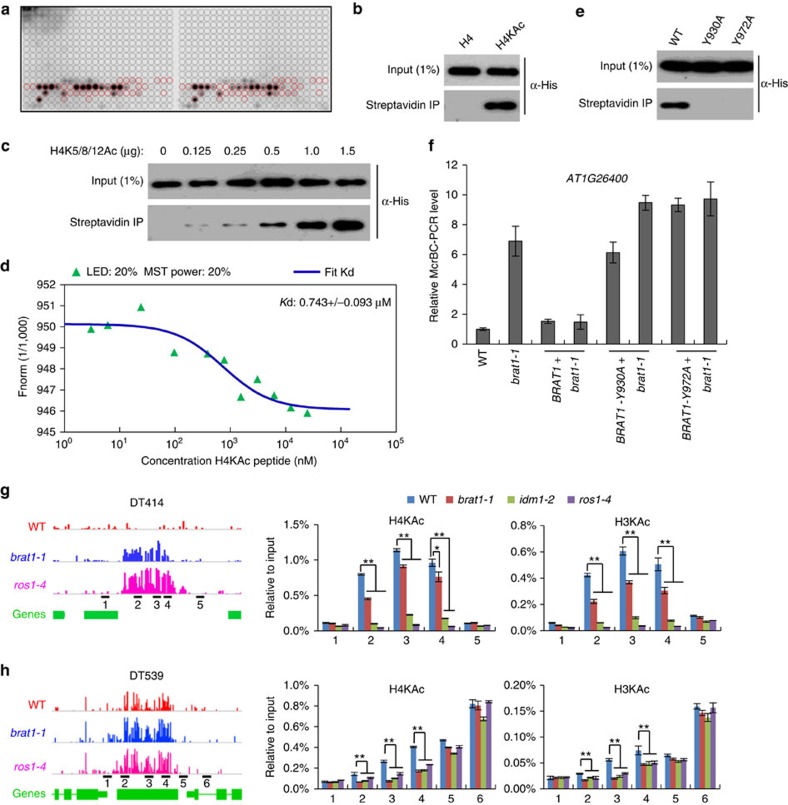
The bromodomain of BRAT1 recognizes acetylated histone H4 and facilitates DNA demethylation. (**a**) The binding of the BRAT1 bromodomain to specific histone marks as determined by a histone peptide array binding assay. Red circles indicate the locations of acetylated histone H4 peptides on the array. (**b**) The interaction of the BRAT1 bromodomain with acetylated histone H4 as determined by pull-down assay. (**c**) Pull-down assay was carried out when an increasing amount of the H4K5/8/12Ac peptide (0, 0.125, 0.25, 0.5, 1.0, 1.5 μg) was added to the binding reaction. (**d**) The interaction between the BRAT1 bromodomain and the acetylated histone H4 peptide was determined by a MST assay. The dissociation constant (*K*d) is shown. (**e**) The effect of the Y930A and Y972A mutations on the interaction between the BRAT1 bromodomain and the acetylated histone H4 was determined by pull-down assay. (**f**) The *BRAT1-Y930A* and *BRAT1-Y972A* transgenes in *brat1-1* fail to complement the DNA hypermethylation phenotypes. (**g**,**h**) Histone H4 and H3 acetylation (ac) levels at *DT414* and *DT539* regions were analysed by ChIP assays. ChIP signals were quantified relative to input DNA. DNA methylation levels and primer localizations are shown on the left. Asterisks indicate statistical significance as determined by Student's *t*-test (**P<0.01; *P<0.05). Error bars are s.d. of three technical replicates in a representative experiment.

BRAT1 is conserved in plants, fungi and animals (Supplementary Figs 13 and 14). It contains an AAA ATPase domain and a bromodomain and is homologous to YTA7, LEX-1 and ATAD2 in *S. cerevisiae*, *C. elegans* and humans, respectively^39^,^41^,^44^. Bromodomain is a conserved module that is present in various nuclear proteins and that binds to acetylated histone peptides including H2A, H2B, H3 and H4 (ref. 28). We purified the bromodomain of BRAT1 (801–1,210 aa) in *E. coli* (Supplementary Fig. 15a,b), and incubated it with a histone peptide array to assess histone binding (Fig. 7a). The binding signals indicate that the BRAT1 bromodomain predominantly interacts with acetylated H4 peptides (Fig. 7a and Supplementary Fig. 16). The interaction of the bromodomain is generally stronger with di- and tri-acetylated H4 peptides than with singly acetylated H4 peptides (Fig. 7a and Supplementary Fig. 16), suggesting a cumulative effect of multiple H4 acetylation on the interaction. With a biotin-labelled H4 pull-down assay, we confirmed that the BRAT1 bromodomain interacts with tri-acetylated H4 peptides (H4K5/8/12Ac) but not with non-acetylated H4 peptides (Fig. 7b). When an increasing amount of the biotin-labelled H4K5/8/12Ac peptide was added to the binding reaction, the bound His-BRAT1 increased (Fig. 7c). Furthermore, we conducted a Microscale Thermophoresis (MST) assay and demonstrated that the BRAT1 bromodomain interacts with the acetylated H4 peptide (Kd=0.743 μM) but not with the non-acetylated H4 peptide (Fig. 7d and Supplementary Fig. 17). The residues Y760 and Y809 in the P/CAF bromodomain, which correspond to Y930 and Y979 in the BRAT1 bromodomain, are necessary for binding to acetylated histone peptides^52^. We mutated Y930 or Y972 to A in the BRAT1 bromodomain and determined whether the mutations affect the interaction of the bromodomain with acetylated H4 peptides. We found that biotin-labelled acetylated H4 peptides pulled down the wild-type BRAT1 bromodomain but not the mutated BRAT1 bromodomain harbouring either of the mutations (Fig. 7e). The results suggest that the BRAT1 bromodomain is responsible for interacting with the acetylated H4 peptide.

To determine whether the bromodomain is required for the function of BRAT1, we transformed the wild-type *BRAT1* construct as well as the mutated *BRAT1* constructs harbouring the Y930 to A or Y972 to A mutation into the *brat1-1* mutant for complementation assays. Western blot analyses indicate that the two mutated *BRAT1* transgenes were expressed as well as the wild-type *BRAT1* transgene (Supplementary Fig. 18). The DNA methylation level of *AT1G26400* is increased in the *brat1* mutant relative to the wild type (Supplementary Fig. 6a). We found that the DNA methylation level of this locus is restored by the wild-type *BRAT1* transgene but not by the mutated *BRAT1* transgenes in the *brat1* mutant (Fig. 7f). Thus, the conserved BRAT1 bromodomain is required for the function of BRAT1 *in vivo*.

Histone acetylation is related to transcriptional activation, but little is known about how histone acetylation contributes to transcriptional activation. The binding of BRAT1 to acetylated H4 suggests that BRAT1 may act as a reader of acetylated H4 to mediate transcriptional activation. IDM1 is a histone H3 acetyltransferase that facilitates the function of ROS1 in active DNA demethylation and transcriptional anti-silencing^18^,^22^. *DT414* and *DT539* are two loci that are hypermethylated and are transcriptionally repressed in *brat1* as well as in *idm1/ros4* and *ros1* (Fig. 7g,h and Supplementary Fig. 10a–c). Our quantitative ChIP (chromatin immunoprecipitation)-PCR analysis indicate that H3 and H4 acetylation is enriched on the two loci (Fig. 7g,h), which is consistent with histone acetylation contributing to active DNA demethylation^18^,^22^.

Acetylation of H3 and H4 at the *DT414* site is dramatically decreased in *idm1* and *ros1*, and to a lesser extend in *brat1* (Fig. 7g). Our whole-genome bisulfite sequencing data indicate that the DNA methylation level of *DT414* is less affected in *brat1* than in *ros1* (Fig. 7g). Thus, the effect of *brat1* on histone acetylation is correlated with its effect on DNA demethylation at this locus. The *DT539* site (the 2nd, 3rd and 4th fragments) is hypermethylated in *brat1*, *idm1* and *ros1* relative to the wild type (Fig. 7h and Supplementary Fig. 10a). At this site, acetylation of H3 and H4 is reduced not only in *idm1* but also in *brat1* and *ros1* (Fig. 7h). Although the *DT539* flanking regions (the 5th and 6th fragments) are enriched with H3 and H4 acetylation (Fig. 7h), the high level of histone acetylation at the *DT539* flanking regions is independent of BRAT1, IDM1 and ROS1, indicating that histone acetylation of these regions may be catalysed by some other histone acetyltransferases rather than by IDM1. The *DT539* flanking regions are not hypermethylated in *brat1* and *ros1* relative to the wild type (Fig. 7h). As previously reported^21^,^24^,^27^, IDM1 is specifically recruited by MBD7 to methylated genomic regions, which is consistent with our finding that IDM1 does not mediate histone acetylation at the unmethylated *DT539* flanking regions (Fig. 7h).

To understand the role of H4Ac in DNA demethylation at the whole-genome level, we downloaded the published acetylated H4K5 ChIP-chip data^53^, and determined whether H4K5 acetylation is enriched in hyper-DMRs of *ros1*, *brat1* and *brp1*. The results indicate that the H4K5Ac level of hyper-DMRs in *ros1* is comparable to that of the whole genome, whereas the H4K5Ac levels of hyper-DMRs are similar in *brat1* and *brp1* and much higher than in *ros1* hyper-DMRs (Supplementary Fig. 19). It is well known that histone acetylation is predominately present in euchromatic genic regions but not in heterochromatic regions. The enrichment of H4K5Ac in hyper-DMRs in *brat1* and *brp1* is consistent with our finding that the hyper-DMRs in *brat1* and *brp1* tend to overlap with genic regions (Fig. 3c). Based on these results we propose that the BRAT1–BRP1 complex may facilitate the function of ROS1 in DNA demethylation at a small set of ROS1 target loci that are present in euchromatic genic regions. Given that histone H4 acetylation of the hyper-DMRs *DT414* and *DT539* is not detectable by the ChIP-chip analysis (Supplementary Data 6), we predict that the histone H4 acetylation levels of the two loci are below the detectability of the ChIP-chip analysis. In the whole-genome histone acetylation analysis, high levels of histone acetylation in unmethylated euchromatic regions may hide the histone acetylation catalysed by IDM1 at methylated heterochromatic regions. Recent studies reported that IDM1 is specifically recruited by the methylated-DNA-binding protein MBD7 to hypermethylated genomic regions and thereby contributes to DNA demethylation^21^,^24^,^27^. Based on our results, we propose that DNA methylation may also be required for BRAT1-mediated prevention of transcriptional silencing in both DNA demethylation-dependent and -independent manners.

## Discussion

BRAT1 is similar to YTA7, LEX-1 and ATAD2 in *S. cerevisiae*, *C. elegans* and humans, respectively^39^,^41^,^42^. However, the similarity is only limited to the ATPase domain and the bromodomain of BRAT1 (Supplementary Fig. 14). YTA7 and LEX-1 are involved in transcription of heterochromatin regions^39^,^40^,^41^. ATAD2 interacts with the E2F transcription factors and is required for cell cycle gene expression and cancer cell proliferation^42^,^43^,^44^. We demonstrate that the BRAT1 bromodomain can bind to acetylated histone and prevents transcriptional silencing at methylated genomic regions. Our results suggest that BRAT1 not only binds to acetylated histone but also contributes to histone acetylation (Fig. 7a–h). Given that histone acetylation is an active chromatin mark, the role of BRAT1 in transcriptional activation is likely caused by its function in histone acetylation. As an acetylated histone-binding protein, BRAT1 may be responsible for maintaining the level of histone acetylation as well as for translating the active histone acetylation code into transcriptional activation. We propose that BRAT1 is recruited to acetylated histone regions by its bromodomain and then creates an appropriate chromatin environment for transcriptional activation. Our study demonstrates that BRAT1 forms a complex with the ATPase domain-containing protein BRP1 and we propose that the BRAT1–BRP1 complex may mediate anti-silencing at methylated genomic regions.

**Figure 8:**
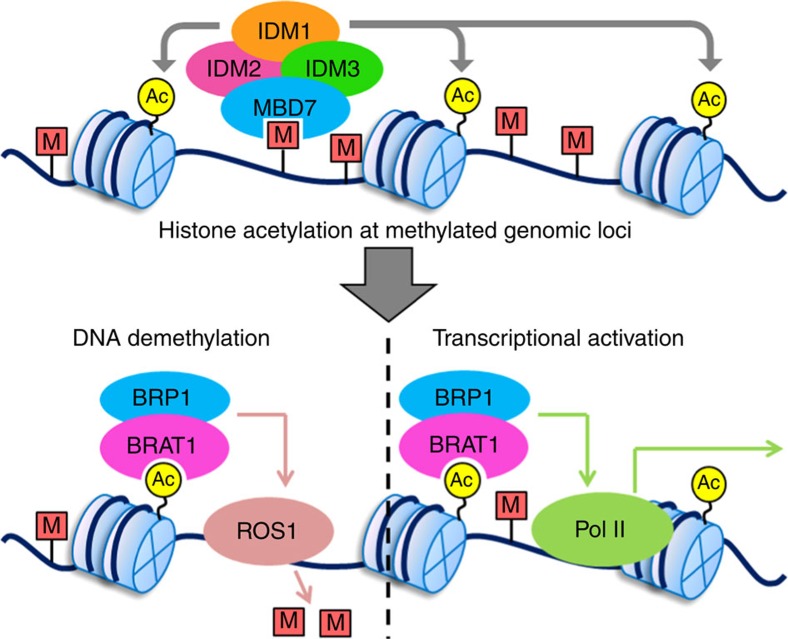
Proposed model for the function of BRAT1 and BRP1 in DNA demethylation and anti-silencing. MBD7 binds to methylated genomic regions and recruits the histone acetyltransferase IDM1 to chromatin, thereby mediating histone acetylation at methylated genomic regions. BRAT1 binds to acetylated histones through its bromodomain and associates with an ATPase domain-containing protein, BRP1 to form a complex. The BRAT1–BRP1 complex may act to facilitate active DNA demethylation by ROS1 and also prevent transcriptional silencing at hypermethylated genomic regions in a DNA demethylation-independent manner.

DNA methylation is actively removed by members of the 5-methylcytosine DNA glycosylase family including ROS1 in *Arabidopsis*^11^,^12^,^14^. The histone acetyltransferase IDM1/ROS4 creates acetylated histones and contributes to DNA demethylation at a subset of loci targeted by ROS1 (refs 18, 22). However, it is unknown how histone acetylation is recognized and then mediates DNA demethylation. Our results suggest that BRAT1 binds to acetylated histone and may contribute to DNA demethylation at a small set of loci targeted by ROS1. Our genetic analyses suggest that BRAT1, IDM1 and ROS1 may mediate DNA demethylation at these loci in the same pathway (Fig. 3f). It is possible that the binding of the BRAT1 bromodomain to acetylated histone may link histone acetylation and DNA demethylation. We propose that the BRAT1–BRP1 complex may act at a downstream step of histone acetylation and create a permissive chromatin environment for DNA demethylation at a subset of ROS1 target loci (Fig. 8). In the BRAT1–BRP1 complex, both BRAT1 and BRP1 contain an ATPase domain, which may provide energy for chromatin structure change.

Although BRAT1 appears to contribute to DNA demethylation at a small subset of ROS1 target loci, DNA demethylation is likely dispensable for most of the BRAT1 anti-silencing function. IDM1 and IDM2 were previously identified as components of the active DNA demethylation pathway^18^,^22^,^23^,^25^. However, both IDM1 and IDM2 are involved in transcriptional activation even when DNA methylation is inhibited in the *ddm1* mutant background^22^,^25^, suggesting that IDM1 and IDM2 may also have a DNA demethylation-independent role in transcriptional activation. Thus, BRAT1, IDM1 and IDM2 likely share a DNA demethylation-independent role. Further studies are required to illustrate how these components are involved in transcriptional activation independent of DNA demethylation. ROS1 was thought to directly remove methylated cytosine by a base excision repair mechanism and act downstream of the histone acetyltransferase IDM1 (refs 13, 14, 18). Interestingly, we found that histone acetylation is affected not only in *brat1* and *idm1* but also in *ros1* (Fig. 7g,h). The results suggest that histone acetylation and DNA demethylation may form a self-reinforcing loop, thereby strengthening the prevention of transcriptional silencing at methylated genomic regions.

IDM1 is a histone H3 acetyltransferase as determined by an *in vitro* assay^18^. Our ChIP-PCR analysis demonstrates that IDM1 contributes to histone acetylation on both H3 and H4 peptides *in vivo* (Fig. 7g,h). Given the positive correlation between histone acetylation and transcriptional activation, the histone acetyltransferase activity of IDM1 may be directly responsible for transcriptional activation. Our study suggests that BRAT1 binds to acetylated histone and prevents transcriptional silencing (Fig. 7a–e and Supplementary Fig. 16). Therefore, BRAT1 may act at a downstream step of the histone acetyltransferase IDM1 and thereby link histone acetylation and transcriptional activation at some methylated genomic regions. Our ChIP-PCR analysis indicates that IDM1 is specifically responsible for histone acetylation at methylated but not unmethylated regions (Fig. 7h), which is consistent with recent studies reporting that IDM1 is recruited by the methylated-DNA-binding protein MBD7 to methylated genomic regions^21^,^24^,^27^. Based on these results, we predict that DNA methylation is required not only for IDM1 but also for the BRAT1–BRP1 complex in the prevention of transcriptional silencing.

## Methods

### Plant materials and mutant screening

The *Arabidopsis* T-DNA insertion lines were obtained from the *Arabidopsis* Biological Resource Center. *Arabidopsis* seedlings were grown on Murashige and Skoog (MS) medium plates with 16 h of light at 23 °C and 8 h of darkness at 20 °C. To screen for mutants in which the transcript level of the RdDM target *AtGP1* is downregulated, total RNA was extracted from 10-day-old seedlings using Trizol reagent (Invitrogen). After contaminating DNA was removed by DNase, 0.5 μg of total RNA was used for RT–PCR to detect the expression of *AtGP1*. PCR primers used in this study are listed in Supplementary Data 7.

For the mutant complementation assays, the genomic DNA of *BRAT1* harbouring the 1,495-bp promoter region was amplified with the primers 1G05910-g-KpnI-F and 1G05910-g-SalI-R, and the PCR fragment was cloned into the *Kpn*I-*Sal*I sites in the modified *pCAMBIA1305* vector with its C-terminal tagged by *3xFlag*. The mutated *BRAT1* sequences were generated by site-directed mutagenesis and cloned into the same *pCAMBIA1305* vector. The genomic DNA of *BRP1* harbouring the 1,746 bp promoter region was amplified with the primers 3G15120-XmaI-F and 3G15120-SpeI-R, and the PCR fragment was cloned into the *Xma*I-*Spe*I sites in the modified *pCAMBIA1305* vector with its C-terminal tagged by *3xMyc*. The above constructs were transformed into the *brat1* or *brp1* mutant by *Agrobacterium* infection. The primers used for plasmids construction and site-directed mutagenesis are listed in Supplementary Data 7.

### Affinity purification and mass spectrometry analysis

Three grams quantity of seedling or flower tissue from *BRAT1-3xFlag* or *BRP1-3xMyc* transgenic plants as well as wild-type plants was used for affinity purification. The tissue was ground in liquid nitrogen and homogenized in 15 ml of Lysis buffer (50 mM Tris, pH 7.6, 150 mM NaCl, 5 mM MgCl_2_, 10% glycerol, 0.1% NP-40, 0.5 mM dithiothreitol (DTT), 1 mM phenylmethylsulphonyl fluoride (PMSF) and one protease inhibitor cocktail tablet per 50 ml, Roche). Following centrifugation, the supernatant was incubated with 100 μl of Anti-Flag M2 Affinity Gel (Sigma, A2220) or Anti-c-Myc Agarose (Sigma, A7470) at 4 °C for 2.5 h. After the resins were washed five times with Lysis buffer, the Flag bead-bound proteins were eluted with 100 μl of 3xFlag peptides (Sigma, F4799), whereas the Myc bead-bound proteins were eluted with 100 μl of 0.1 M ammonium hydroxide. The eluted proteins were run on a 10% SDS–polyacrylamide gel electrophoresis (SDS–PAGE) gel and then subjected to silver staining with the ProteoSilver Silver Stain Kit (Sigma, PROT-SIL1). The mass spectrometric analysis was performed according to Zhang *et al*.^54^ Briefly, proteins on SDS–PAGE gels were de-stained and digested in-gel with trypsin at 37 °C overnight. The digested peptides were eluted on a capillary column and sprayed into an LTQ mass spectrometer equipped with a nano-ESI ion source for analysis (Thermo Fisher Scientific).

### Co-IP and nuclear fractionation

For co-IP analysis between BRAT1 and BRP1 or ROS1, the *BRAT1-Flag* transgenic plants were crossed to *BRP1-Myc* or *ROS1-Myc* transgenic plants. A 1-g quantity of tissue from parental lines as well as from F1 plants was ground in liquid nitrogen and homogenized in 5 ml of Lysis buffer. Following centrifugation, the supernatant was incubated with 50 μl of Anti-Flag M2 Affinity Gel at 4 °C for 2.5 h. The resins were washed five times with Lysis buffer, and the bead-bound proteins were eluted with 3xFlag peptides. The input and eluted proteins were run on a 7.5% SDS–PAGE gel for western blotting analysis. Nuclear fractionation was performed according to the study by Zhang *et al*.^55^ Briefly, 1 g of 10-day-old seedlings were ground in liquid nitrogen and homogenized in 5 ml of Honda buffer (20 mM HEPES-KOH, pH 7.4, 0.44 M sucrose, 1.25% ficoll, 2.5% Dextran T40, 10 mM MgCl_2_, 0.5% Triton X-100, 5 mM DTT, 1 mM PMSF, Proteinase inhibitor cocktail). The homogenate was filtered through two layers of Miracloth, followed by centrifugation at 1,500*g* for 5 min. The pellet was washed for two times with Honda buffer and one time with 1 × PBS/1 mM EDTA. Then, the pellet was resuspended in 0.5 ml of prechilled glycerol buffer (20 mM Tris-HCl, pH 7.9, 75 mM NaCl, 0.5 mM EDTA, 0.85 mM DTT, 50% glycerol, 0.125 mM PMSF, Proteinase inhibitor cocktail, 10 mM β-mercaptoethanol), to which 0.5 ml cold nuclei lysis buffer (10 mM HEPES, pH 7.6, 1 mM DTT, 7.5 mM MgCl_2_, 0.2 mM EDTA, 0.3 M NaCl, 1 M Urea, 1% NP-40, 0.5 mM PMSF, Proteinase inhibitor cocktail, 10 mM β-mercaptoethanol) was added and gently vortexed for two times, each for 2 s, then incubated on ice for 2 min, and centrifuged at 14,000 r.p.m. at 4 °C for 2 min to separate the chromatin fraction and the nucleoplasmic fraction. The proteins were extracted from the nucleus, the nucleoplasm and the chromatin, and then analysed by western blotting. Full scans of the agarose gels and western blots are shown in Supplementary Fig. 20.

### Histone peptide array and pull-down assay

The coding sequence of C-terminal BRAT1 (801–1,210 aa), including the bromodomain, was amplified and cloned into the *pET28a* vector. The bromodomain mutation sites were introduced by site-direct mutagenesis. These constructs were transformed into *E. coli* BL21 for fusion protein expression. The His-Tag fusion proteins were purified with Ni-NTA His Bind Resin (Novagen). MODified Histone Peptide Array (Active Motif) was used to screen the specific histone marks for BRAT1 binding. After the array was blocked at room temperature (RT) for 1 h in 20 ml of TTBS buffer (10 mM Tris-HCl, pH 7.5, 150 mM NaCl and 0.05% Tween-20) containing 5% non-fat dried milk, it was washed three times (5 min each time) in TTBS buffer at RT and then incubated with 50 μg of purified His fusion C-terminally BRAT1 protein overnight at 4 °C in 10 ml of binding buffer (50 mM HEPES, pH 7.5, 50 mM NaCl, 5% glycerol, 0.4% BSA, 2 mM DTT). Then, the array was washed three times (5 min each time) in 20 ml of TTBS buffer at RT before it was incubated with anti-His antibody in TTBS buffer for 2 h at RT. Finally, the array was washed five times with TTBS, developed by Amersham ECL Prime, and exposed to X-ray film to capture images. The image was analysed using Active Motif Array Analyze software.

For pull-down assay, the biotinylated histone H4 (P0058), H4K5AcK8AcK12Ac (P0072) peptide were purchased from EpiCypher. Each peptide (1 μg) was incubated with 5 μg of His-Tag fusion C-terminally BRAT1 protein in binding buffer at 4 °C for 4 h. Then, the mixture was incubated with 30 μl of Streptavidin MagneSphere Paramagnetic Particles (Promega, Z5481) for 2 h with rotation. The beads were washed four times, and then eluted with SDS sample buffer. The input and eluted proteins were run on an SDS–PAGE gel for western blotting analysis with His antibody.

### MST-binding assay

For MST-binding assays, the purified BRAT1 C-terminal domain was labelled with the Monolith NT Protein Labeling Kit RED-NHS. The labelled protein was used at a concentration of 100 nM in phosphate-buffered saline (pH 7.6) containing 0.05% Tween-20. The concentration of the non-acetylated H4 peptide and the acetylated H4 peptide are ranged from 3 nM to 25 μM. The reaction is aspirated by capillary forces into the MST glass capillary. MST was measured on a NanoTemper Monolith NT.115 (20% light emitting diode power; 20% laser power). A dissociation constant (Kd) was calculated to determine the binding affinity.

### RNA deep sequencing and data analysis

Total RNA was extracted from 0.2 g of 10-day-old *Arabidopsis* seedlings with Trizol reagent (Invitrogen) and sent to BGI (Wuhan, China) for library preparation and Illumina sequencing. For library preparation, 3 μg RNA was purified from total RNA using poly-T oligo-attached magnetic beads. The NEBNext Ultra RNA Library Prep Kit for Illumina (NEB, USA) was used to generate libraries according to the manufacturer's protocol. The libraries were sequenced on an Illumina Hiseq 2500 platform. Reads were mapped to the TAIR10 genome with Tophat (v2.0.6) allowing up to two mismatches. Only reads mapped unique to the genome were used for further analysis. The differential expression of genes and TEs was analysed with the Cufflinks (v2.0.1) package.

### Whole-genome bisulfite sequencing and data analysis

Genomic DNA was extracted from 10-day-old *Arabidopsis* seedlings with the DNeasy Plant Mini Kit (Qiagen, 69104) and sent to BGI (Shenzhen, China) for bisulfite treatment, library preparation and Illumina sequencing. For library preparation, 5 μg of genomic DNA was sonicated to 100–300 bp and end-repaired. Cytosine-methylated adaptors from Illumina were ligated according to the manufacturer's instructions. Unmethylated cytosine residues were converted to uracils using the EpiTect bisulfite kit (Qiagen). Libraries were prepared for sequencing according to the manufacturer's (Illumina) instructions and sequenced on an Illumina Hiseq 2500 platform. For data analysis, reads were mapped to the TAIR10 genome by Bismark (v0.10.0) allowing up to two mismatches. DNA methylation was calculated when cytosine sites had at least threefold coverage. DNA methylation levels in every 50-base-pair bin were compared between wild-type and mutant plants using Fisher's exact test. The differentially methylated bins were combined to generate differently methylated regions when their gap size was ≤50 base pairs. ‘Overlap' is called when there are ≥50 bp overlaps between hyper-DMRs. Overlaps between DMRs in *brat1*, *brp1* and *ros1* were assessed by hypergeometric test. Expected number of overlapped DMRs by chance=(A*B)/*N*, where *A*=number of DMRs in mutant 1; *B*=number of DMRs in mutant 2; *N*=total number of bins in *Arabidopsis*.

### DNA methylation analysis at individual loci

DNA methylation of individual genomic loci was analysed by bisulfite sequencing and chop-PCR. For bisulfite sequencing, 2 μg of genomic DNA was used according to the protocol of the EpiTect bisulfite kit (Qiagen). The bisulfate-treated DNA was amplified, and the amplification product was cloned into the *pGEM-T* vector (Promega) for sequencing. The DNA methylation levels at CG, CHG and CHH sites were separately calculated. For chop-PCR, genomic DNA was cleaved with the methylation-sensitive restriction enzymes *Hha*I and *Bst*UI or the methylation-dependent restriction enzyme McrBC, and the products were subjected to PCR.

### Analysis of RNA transcripts at individual loci

Total RNA was extracted from 10-day-old *Arabidopsis* seedlings with Trizol reagent (Invitrogen). After contaminating DNA was removed by DNase I (Invitrogen), 1 μg of total RNA was used for reverse transcription using both oligo dT and random primers. The reverse transcription products (cDNA) were diluted fivefold, and 5 μl of the diluted cDNA was used for quantitative PCR in a 20-μl reaction mixture. For quantitative RT–PCR, SYBR Premix Ex Taq II (Tli RNaseH Plus) (RR420A; Takara) was used on an Applied Biosystems 7500 Fast real-time PCR system. The results presented were based on at least three replications.

### ChIP assay

The levels of histone H4KAc or H3KAc on chromatin were determined by ChIP assay. Three grams of 10-day-old seedlings were fixed in 0.5% formaldehyde under vacuum. Chromatin was extracted with Honda Buffer (20 mM HEPES-KOH, pH 7.4, 0.44 M sucrose, 1.25% ficoll, 2.5% Dextran T40, 10 mM MgCl_2_, 0.5% Triton X-100, 5 mM DTT, 1 mM PMSF, Proteinase inhibitor cocktail) and sonicated. The chromatin was then incubated with 2 ug H4KAc antibody (Millipore, 06–866) or H3KAc antibody (Millipore, 06–599) at 4 °C overnight. The chromatin bound by H4KAc or H3KAc was purified and used for PCR with sequence-specific primers listed in Supplementary Data 7.

### Analysis of enrichment of H4K5 acetylation on hyper-DMRs

The histone H4K5Ac ChIP-chip data were generated by a previous study^53^. We downloaded the primary data from GEO with accession GSM543332. H4K5ac intensity peaks were searched using the MAT package with *P* value<1E-05. Coordinate was remapped from TAIR7 to TAIR10 using the NCBI genome remapping service. H4K5Ac regions were separately compared with hyper-DMRs in *ros1*, *brat1* and *brp1*. The enrichment of H4K5Ac on different hyper-DMRs was determined.

## Additional information

**Accession codes:** All high-throughput sequencing data generated in this study have been deposited in GEO with accession GSE67799.

**How to cite this article:** Zhang, C.-J. *et al*. The *Arabidopsis* acetylated histone-binding protein BRAT1 forms a complex with BRP1 and prevents transcriptional silencing. *Nat. Commun.* 7:11715 doi: 10.1038/ncomms11715 (2016).

## Supplementary Material

Supplementary Information Supplementary Figures 1-20 and Supplementary Tables 1-2

Supplementary Data 1 List of homozygous T-DNA mutants used in this study

Supplementary Data 2 Hypermethylated regions in ros1-4

Supplementary Data 3 Differentially expressed TEs (log2(fold_change)>1 or < -1, p_value < 0.05) in brat1-1 relative to Col-0

Supplementary Data 4 Up-regulated TEs (log2(fold_change)>1, p_value < 0.01) in nrpe1-11 relative to Col-0

Supplementary Data 5 Down-regulated TEs (log2(fold_change)< -1, p_value < 0.01) in nrpe1-11brat1-1 relative to nrpe1-11

Supplementary Data 6 List of H4K5Ac regions as determined by the ChIP-chip data

Supplementary Data 7 List of primers used in this study

## Figures and Tables

**Table 1:** List of BRAT1-Flag and BRP1-Myc co-purified proteins.

Accession number	Annotation	Protein	Mascot score	MW (Da)	Spectra count	Unique peptides
*BRAT1-Flag purified proteins*						
IPI00542285	AT1G05910	BRAT1	2,639	133,701	72	45
IPI00537350	AT3G15120	BRP1	1,236	215,740	30	26
						
*BRP1-Myc purified proteins*						
IPI00537350	AT3G15120	BRP1	1,664	215,740	40	33
IPI00542285	AT1G05910	BRAT1	1,489	133,701	37	28

The purified proteins were subjected to mass spectrometric analysis. Mascot score of each protein is shown.
