# Supplementary material for: The Arabidopsis acetylated histone-binding protein BRAT1 forms a complex with BRP1 and prevents transcriptional silencing
Source: Nat Commun. 2016 Jun 7;7:11715. doi: 10.1038/ncomms11715 (PMC4899616; doi:10.1038/ncomms11715)
Supplement: Supplementary Information — Supplementary Figures 1-20 and Supplementary Tables 1-2 [file ncomms11715-s1.pdf]

Supplementary Figure 1

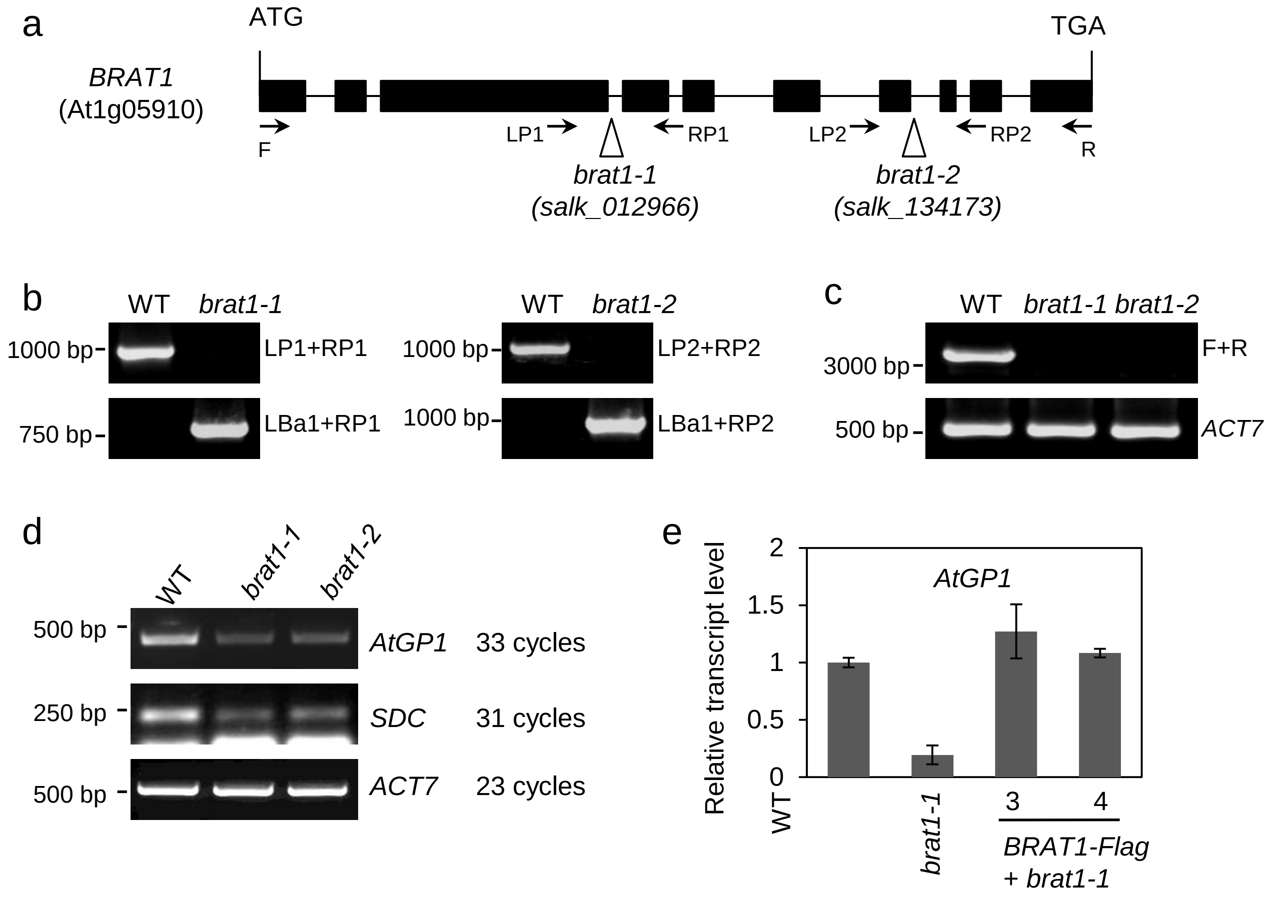

Supplementary Fig. 1. Characterization of T-DNA insertion mutations in *BRAT1*. (**a**) Schematic representation of the T-DNA insertions in the *BRAT1* gene. Black boxes and solid lines represent exons and introns, respectively. The arrows represent PCR primers used in genotyping and RT-PCR analysis. (**b**) Genotyping analysis of *brat1* T-DNA mutant alleles. (**c**) RT-PCR analysis of *BRAT1* gene expression in the wild type and *brat1* mutants. (**d**) The RNA transcripts of *AtGP1*, and *SDC* were detected by RT-PCR in WT (wild-type) and *brat1* mutants. The *ACT7* was amplified as an internal control. (**e**) The expression of *AtGP1* was detected by quantitative RT-PCR in the wild-type Col-0, *brat1-1*, and two representative *BRAT1-Flag* transgenic lines in the *brat1-1* mutant background. Error bars represent s.d. of three biological replicates.

Supplementary Figure 2

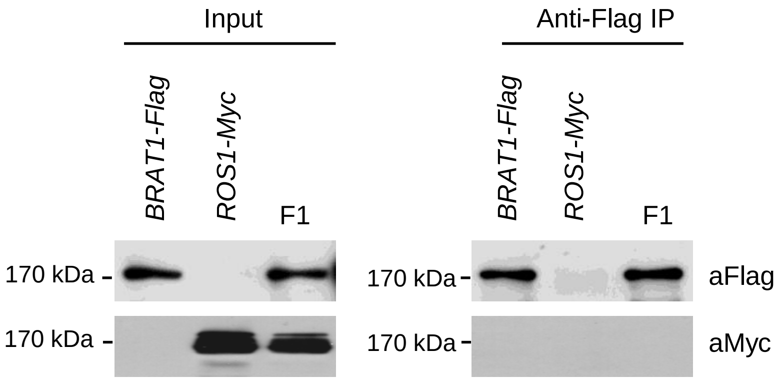

Supplementary Fig. 2. BRAT1 and ROS1 do not interact. The interaction between BRAT1-Flag and ROS1-Myc was determined by co-IP. *BRAT1-Flag* transgenic plants were crossed to *ROS1-Myc* transgenic plants, and the F1 plants expressing both fusion proteins were subjected to co-IP.

Supplementary Figure 3

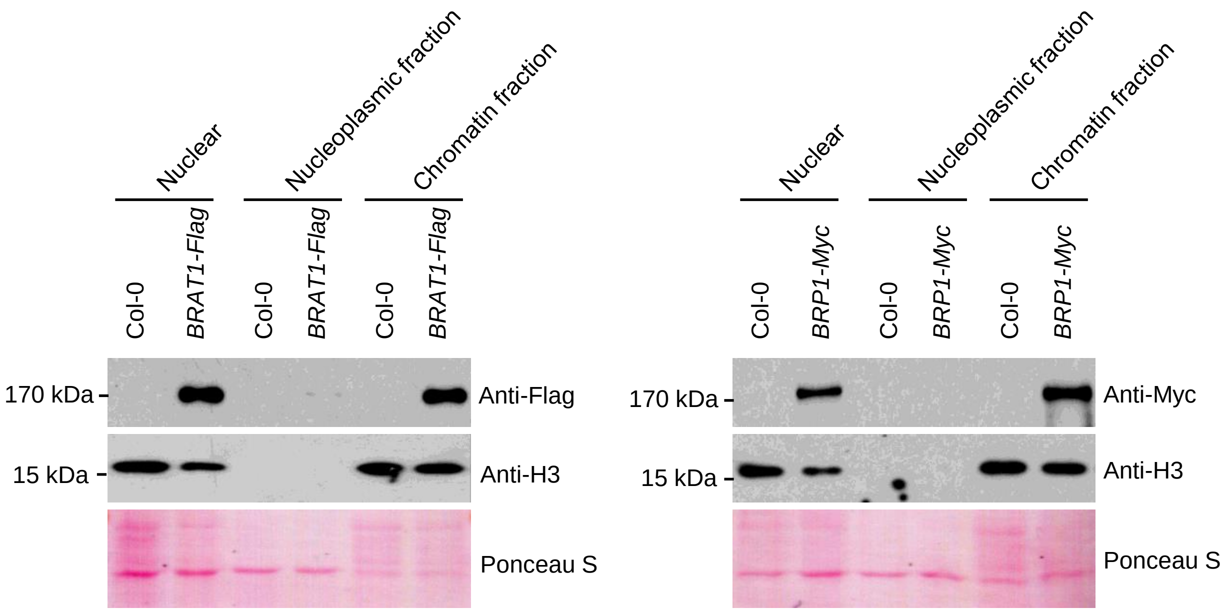

Supplementary Fig. 3. Western blot analysis of the subnuclear localization of BRAT1-Flag and BRP1-Myc. The nuclear proteins were fractionated into the nucleoplasmic fraction and the chromatin fraction for the wild-type Col-0 and the transgenic seedlings. The proteins in the different fractions were analyzed by Western blotting with histone H3, Flag, and Myc antibodies. Ponceau S staining is shown as a loading control.

Supplementary Figure 4

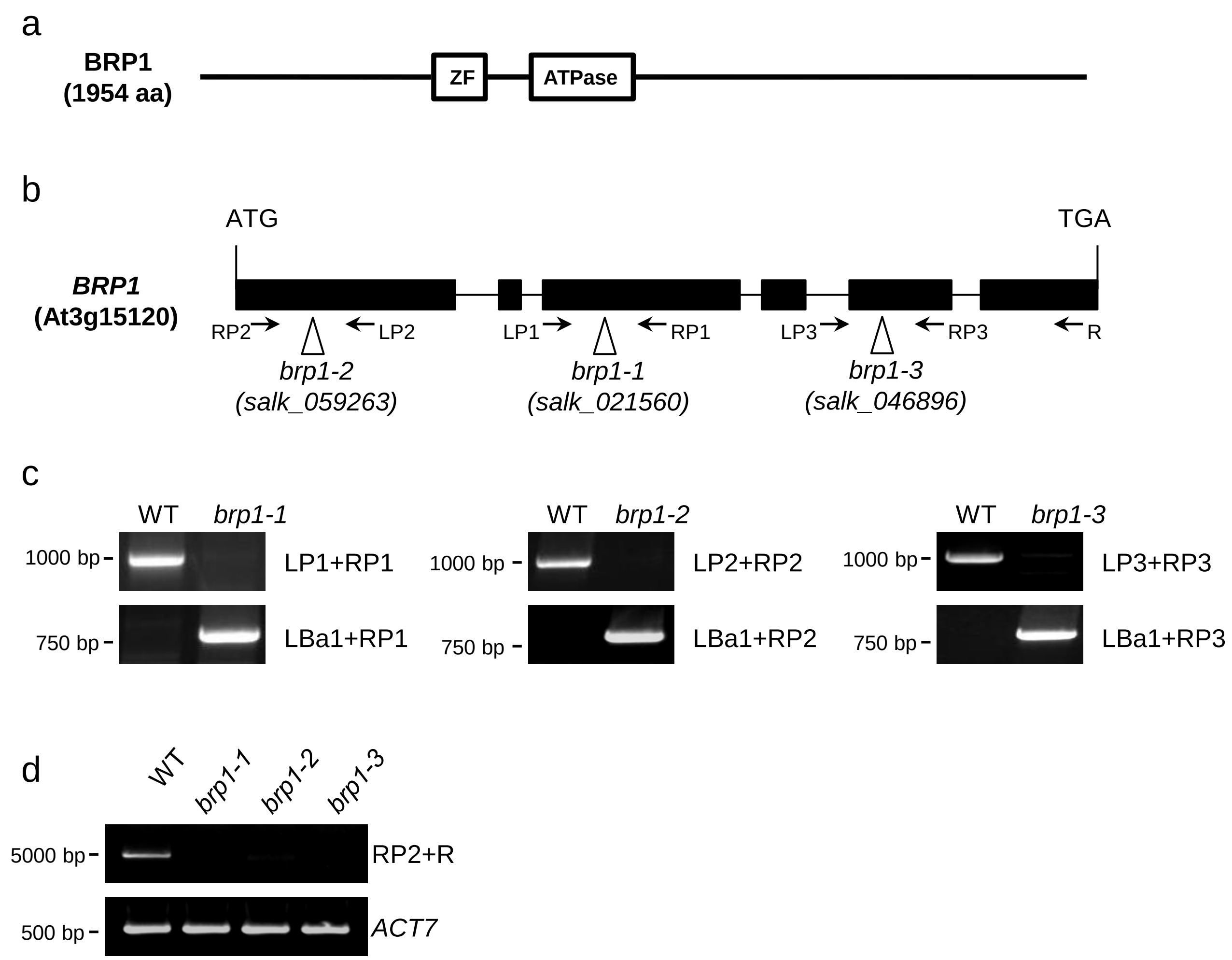

Supplementary Fig. 4. Characterization of *brp1* T-DNA insertion mutants. (**a**) Diagram of the BRP1 protein. (**b**) Schematic representation of the T-DNA insertions in the *BRP1* gene. Exons and introns of the gene are represented by black boxes and solid lines, respectively. (**c**) Genotyping analysis of the individual *brp1* mutants *brp1-1*, *brp1-2*, and *brp1-3*. (**d**) The expression of *BRP1* as determined by RT-PCR in the wild type and the three individual *brp1* mutant alleles. *ACT7* is an internal control.

Supplementary Figure 5

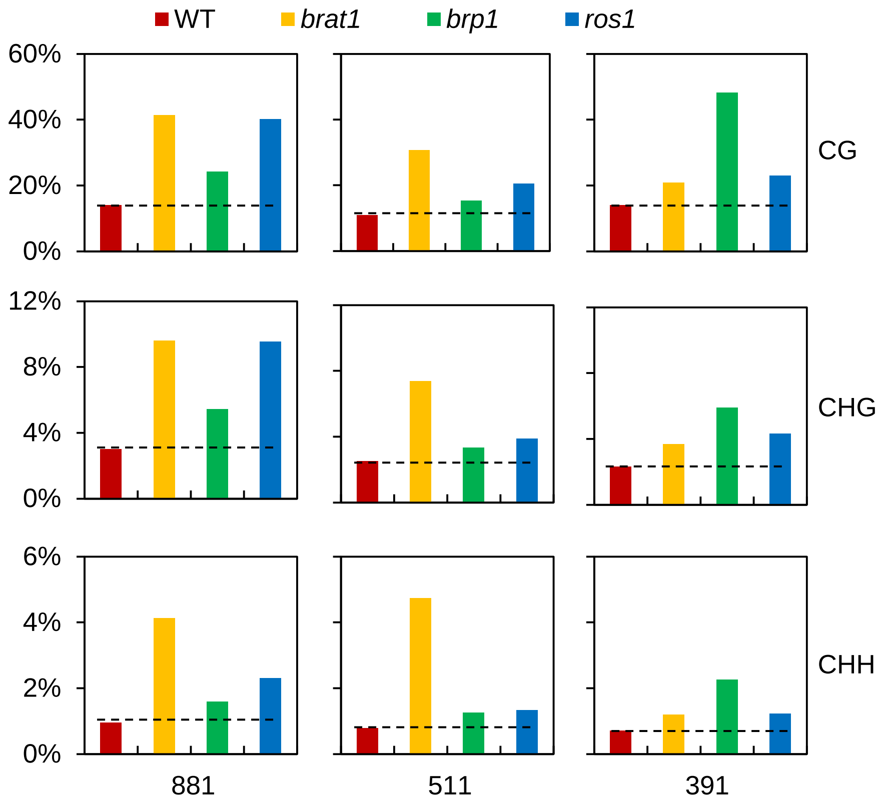

Supplementary Fig. 5. The average methylation levels of the *brat1* and *brp1* targets. The average methylation levels of CG, CHG, and CHH were calculated based on the whole-genome bisulfite sequencing data in wild type, *brat1*, *brp1*, and *ros1*. The numbers “881”, “511”, and “391” represent hyper-DMRs in *brat1*, *brat1*-specific hyper-DMRs, and *brp1*-specific hyper-DMRs, respectively, which are related to Figure 3a.

Supplementary Figure 6

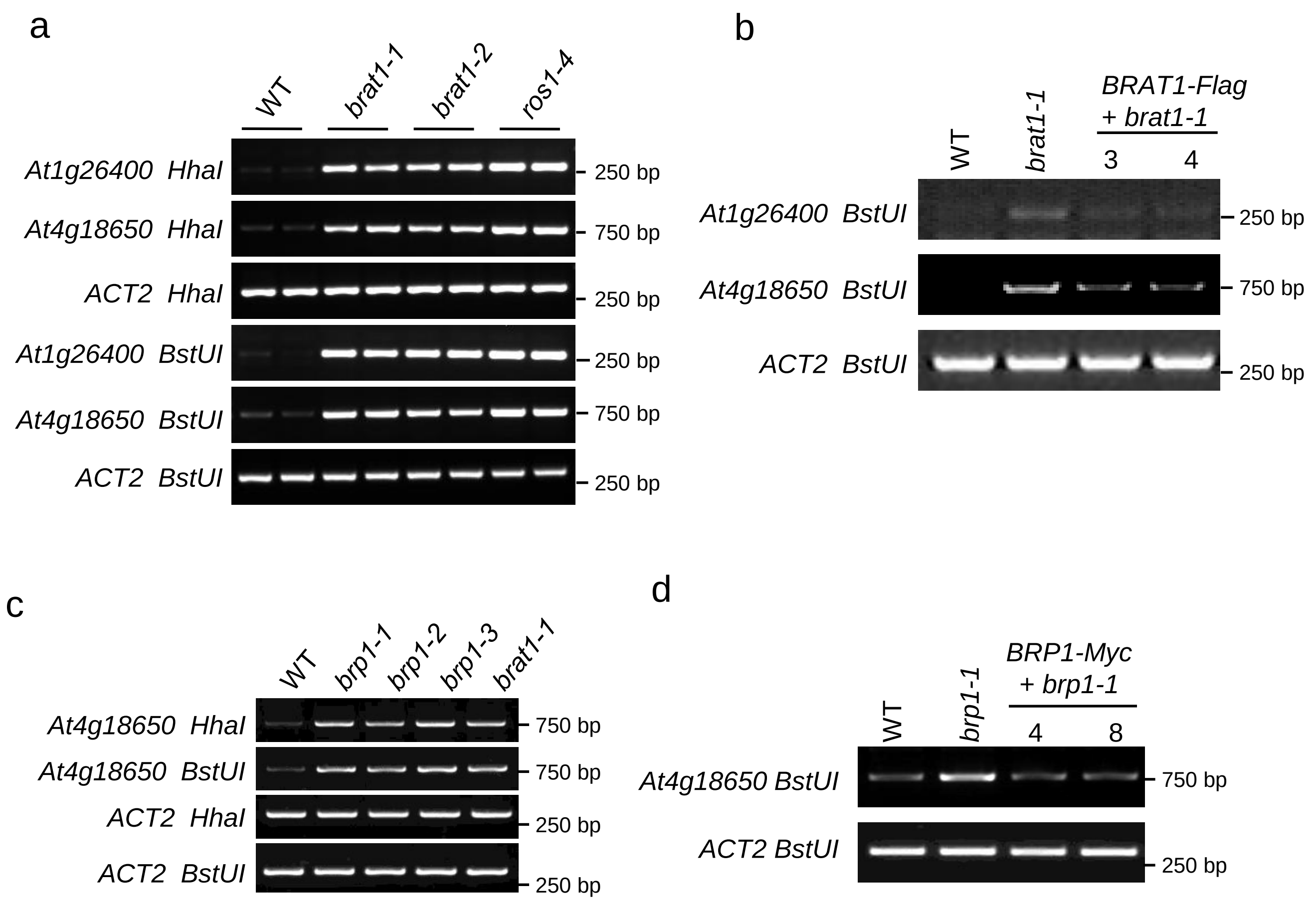

Supplementary Fig. 6. Effect of *brat1* and *brp1* on DNA methylation of *AT1G26400* and *AT4G18650*. (**a**) DNA methylation of *AT1G26400* and *AT4G18650* was detected by chop-PCR in the wild type and the *brat1-1*, *brat1-2*, and *ros1-4* mutants. Genomic DNA was cleaved by the DNA methylation-sensitive restriction enzymes HhaI and BstUI. (**b**) DNA methylation of AT1G26400 and AT4G18650 was determined by chop-PCR in two representative *BRAT1-Flag* transgenic lines for complementation analyses. *BsTUI* is a DNA methylation-sensitive restriction enzyme.(**c**) DNA methylation of *AT4G18650* was detected by chop-PCR in the wild type, *brp1*, and *brat1*. (**d**) The complementation of *brp1* was determined by chop-PCR in two representative *BRP1-Myc* transgenic lines.

Supplementary Figure 7

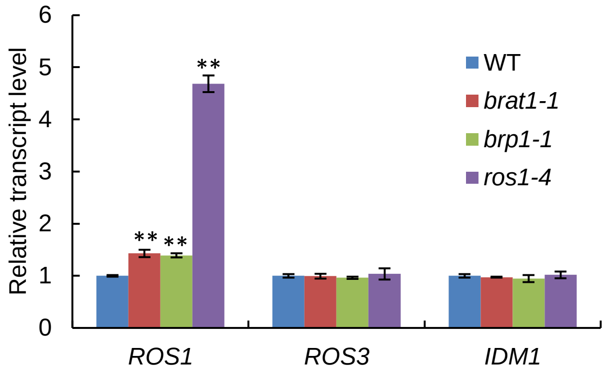

Supplementary Fig. 7. The effect of *brat1*, *brp1* and *ros1* on the expression of *ROS1*, *ROS3*, and *IDM1*. The RNA transcripts of *ROS1*, *ROS3*, and *IDM1* were detected by quantitative RT-PCR in wild type, *brat1*, *brp1*, and *ros1*. *ACT7* was amplified as an internal control. Asterisks indicate statistical significance as determined by Student's t test (**P<0.01). Error bars represent s.d. of three biological replicates.

Supplementary Figure 8

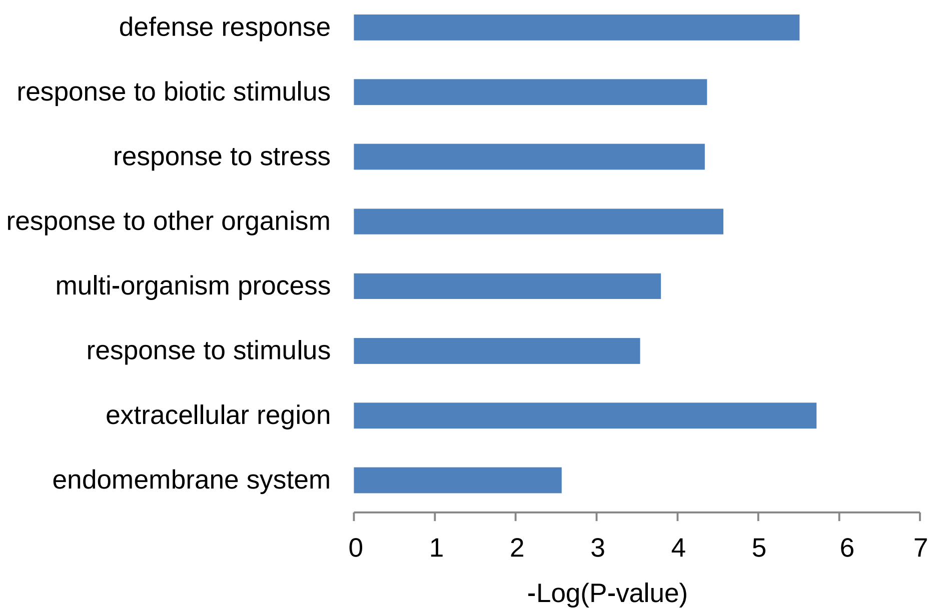

Supplementary Fig. 8. Gene Ontology (GO) analysis of differentially expressed genes in *brat1* mutant. Statistical values of gene enrichment in the indicated biological processes are represent with the length of bars. The biological processes are listed only when their genes are significantly (P<0.01; Fisher's exact test) enriched.

Supplementary Figure 9

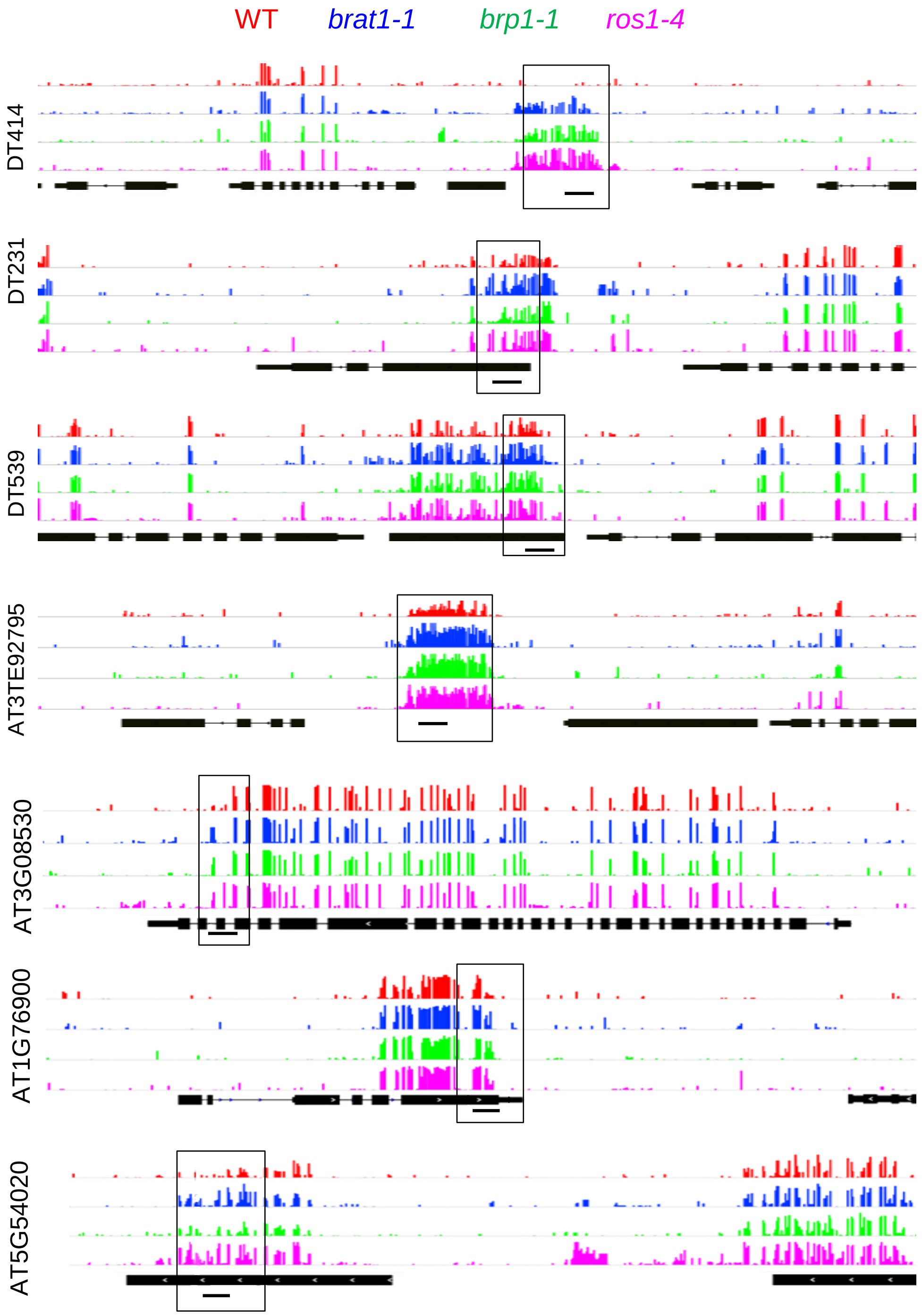

Supplementary Fig. 9. Snapshots showing DNA methylation patterns of hyper-DMRs in the wild type, *brat1-1*, *brp1-1*, and *ros1-4*. Boxes indicate hypermethylated regions in *brat1*, *brp1*, and *ros1* relative to the wild type. Small bars indicate regions that are subjected to PCR-based DNA methylation analyses and quantitative RT-PCR in Supplementary Figure 10a-d.

Supplementary Figure 10

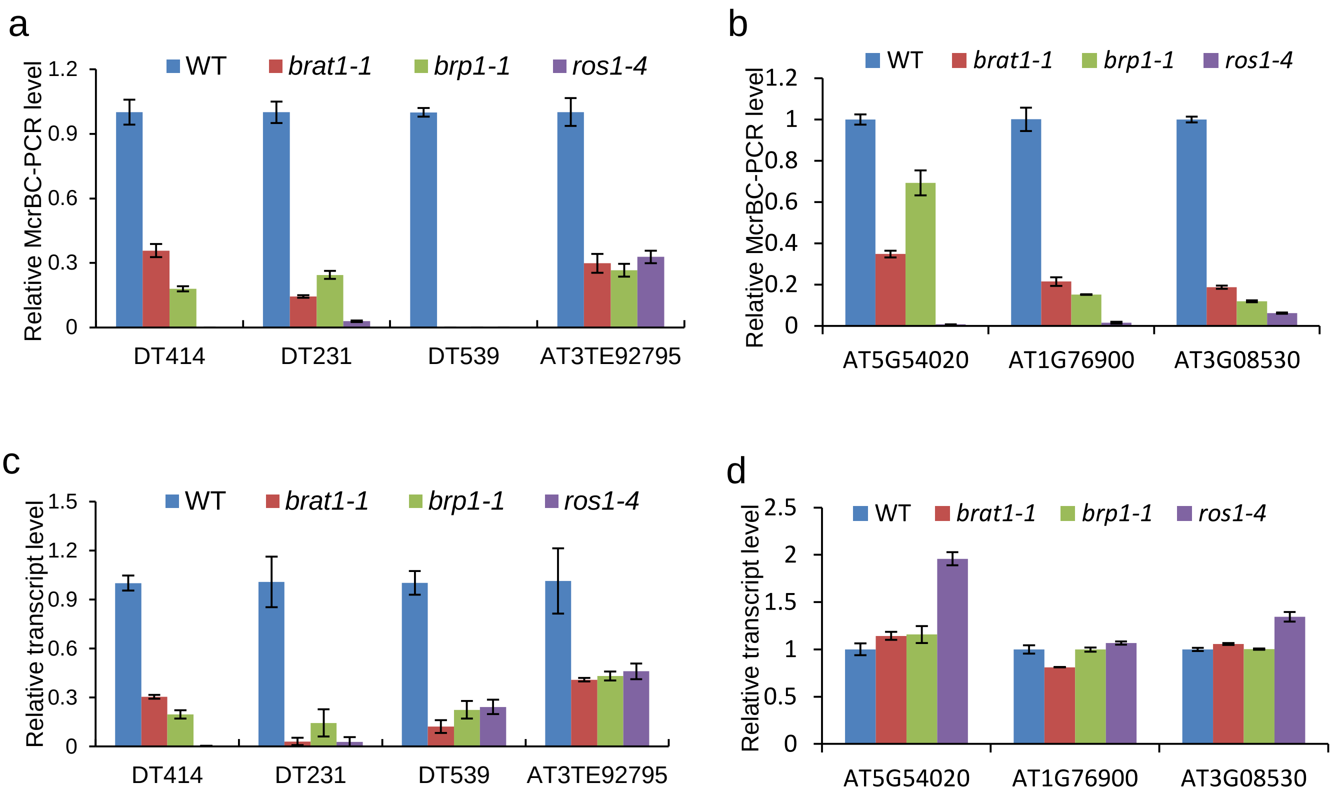

Supplementary Fig. 10. Transcript levels were affected at a subset of hyper-DMRs in *brat1*, *brp1*, and *ros1*. (**a,b**) DNA methylation levels of the hyper-DMRs indicated in Supplementary Fig. 9 were evaluated by quantitative chop-PCR in the wild type, *brat1-1*, *brp1-1*, and *ros1-4*. For PCR-based DNA methylation analyses, genomic DNA was cleaved by the methylation-dependent restriction enzyme McrBC followed by quantitative PCR. The DNA methylation levels were normalized using *ACT2* as an internal control. (**c,d**) RNA transcript levels of the hyper-DMRs were evaluated by quantitative RT-PCR in the wild type, *brat1-1*, *brp1-1*, and *ros1-4*. The expression of *ACT7* was used as an internal control. Error bars are s.d. of three technical replicates of a representative experiment.

Supplementary Figure 11

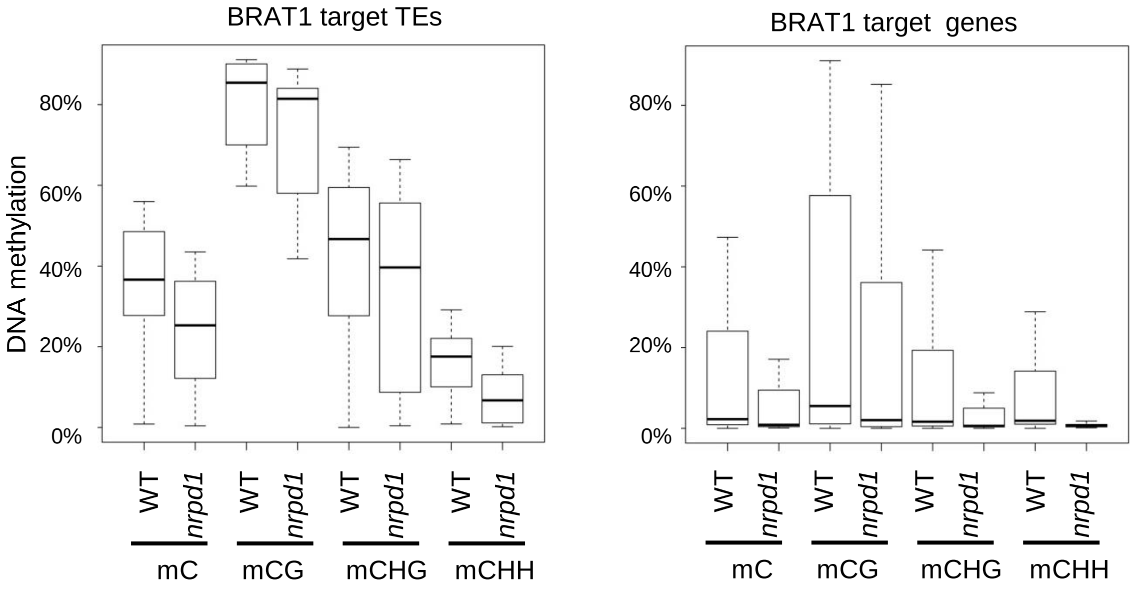

Supplementary Fig. 11. Effect of *nrpd1* on DNA methylation of transcriptionally down-regulated TEs and genes in the *brat1* mutant. The published whole-genome DNA methylation data (Stroud et al., 2013) were used to analyze the DNA methylation levels of transcriptionally down-regulated TEs and genes identified in the *brat1* mutant. The DNA methylation levels of these down-regulated TEs and genes identified in the *brat1* mutant were analyzed in the wild type and the *nrpd1* mutant. The TEs and genes used for box plotting are shown in Supplementary Data 3.

Supplementary Figure 12

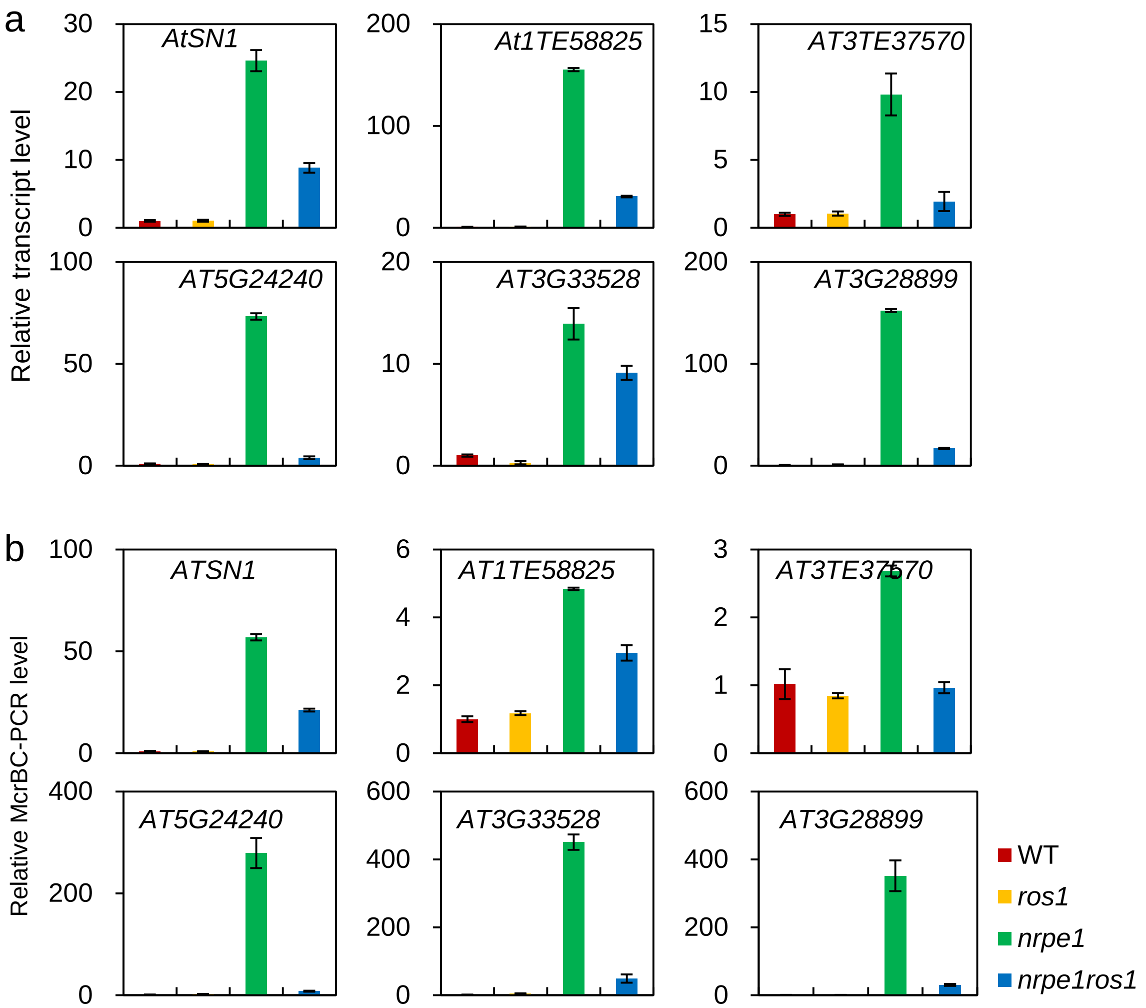

Supplementary Fig. 12. ROS1 contributes to the expression of RdDM target loci in the *nrpe1* mutant. RNA transcripts (**a**) and DNA methylation (**b**) of *AtSN1*, *AT1TE58825*, *AT3TE37570*, *AT5G24240*, *AT3G33528,* and *AT3G28899* were evaluated by quantitative RT-PCR and chop-PCR, respectively, in wild type, *ros1*, *nrpe1*, and *nrpe1ros1*. For RT-PCR, *ACT7* was amplified as an internal control. For chop-PCR, genomic DNA was cleaved by the methylation-dependent restriction enzyme McrBC followed by quantitative PCR. Error bars represent s.d. of three technical replicates of a reprsentative experiment.

Supplementary Figure 13

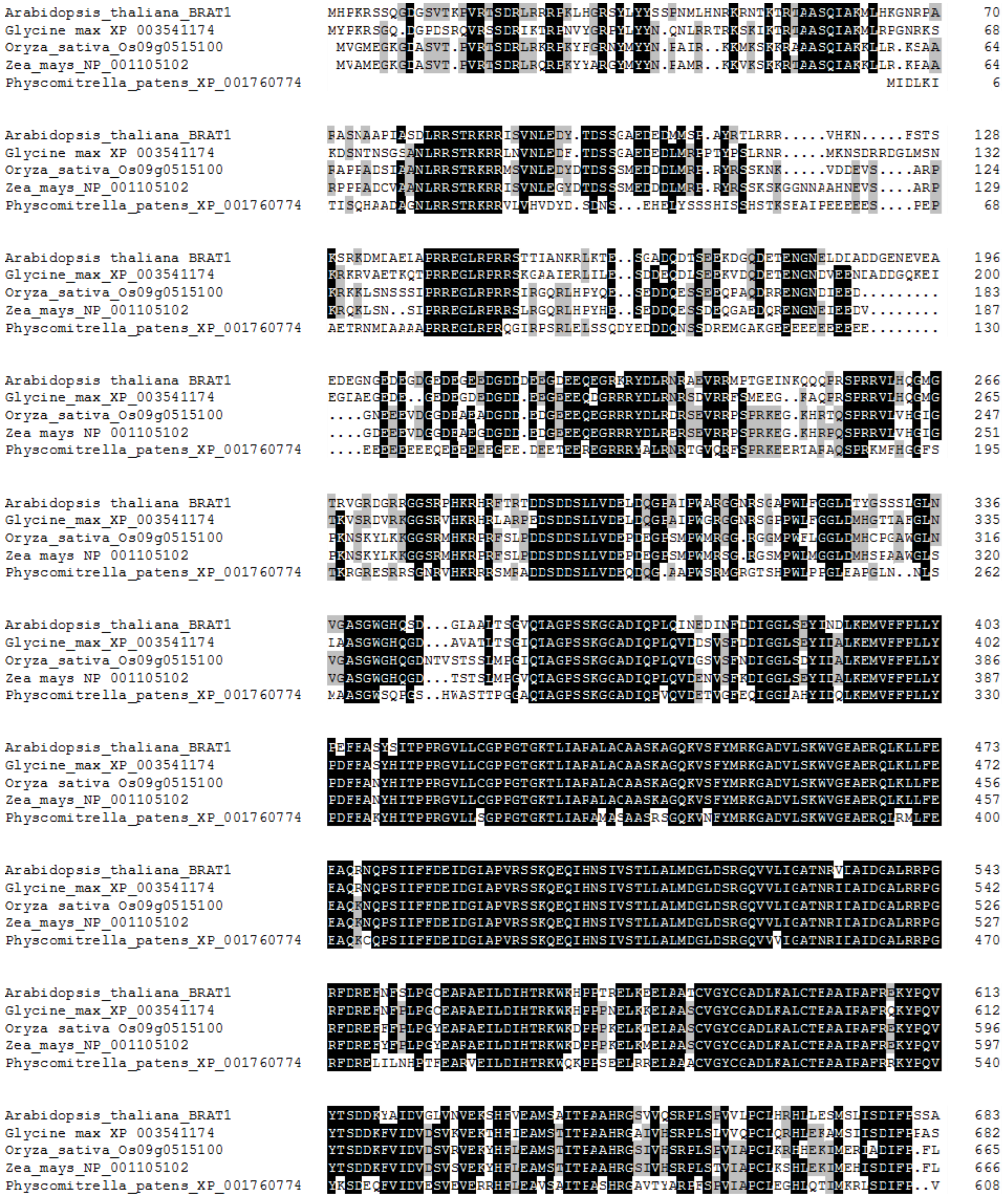

```
Arabidopsis_thaliana_BRAT1            TSSELTKLSILTFGSAIPLVYRPRLLLLGGEGVGLDHLGFAILHELEKFPIHSLGLPSLLSDPGAKTPEE    753
Glycine max XP 003541174              ITSELTKLSMLSYGSAIPLVYRPRLMLCGGEGTGLDHLGFAVLHELEKFPVHSLGLPSLLSDPSAKTPEE    752
Oryza_sativa_Os09g0515100             SSVDVSKFSALSYGSSIPLVYRPRLLMCGGVSVGLDHVGFAVLHELEKFSVHSLGLPSLLSDPSAKTPEE    735
Zea_mays_NP_001105102                 SSIDFSKFSALSYGSSIPLVYRPRLLICGGESVGLDHVGFAVLHELEKFSVHSLGLPSLLSDPSAKTPEE    736
Physcomitrella_patens_XP_001760774    MNRDISNTRLFGLGCSAPLVYRPKFLLCGREGAGLDHVGFAVLHELERFPVHSLGLPSLLSDPSAKSPEE    678

Arabidopsis thaliana BRAT1            ALVHIFSEARRTTPSILYIPMFNNWWENAHEQLFAVFLTLLEELPSNLPILLLATSYGELSDMEEQ..SV    821
Glycine_max_XP_003541174              ALVHIFGEARRTTPSILYLPQFDVWWETAHEQLFAVLLTLLEELPSDLPILLLGTSSVTIAEVEEVPTSI    822
Oryza_sativa_Os09g0515100             ALVHIFGEARRTTPSILYLPQFHLWWDIAHEQLFAVLLTLLNELPSNLPVLLLGTSSVAFGDLEEECASI    805
Zea mays NP 001105102                 ALVHIFGEAKRTTPSILYIPQFHLWWDIAHEQLFAVLLTLLNELPSNLPVLLLGTSSVVFTDLEEECASI    806
Physcomitrella_patens_XP_001760774    ALVHIFGEARRNTPSILYLPQLHLWWDIAHDQLFAVLKMLLGDLPSDLPVLFLATACVCGKDLDEDIFAL    748

Arabidopsis thaliana BRAT1            FDNRSVYTVDKPSSEDRSLFFDRLIEAALSVISG.LNGKPDGPQPLPELPKVPKEPTGPKFAEVFAKVEA    890
Glycine_max_XP_003541174              FPHRSIYKVNMPCAKDRTLFFNLLIEAAMSILLEGINKKSQIAGCLPELPKAPKIASGPKVSELFAKVEA    892
Oryza sativa Os09g0515100             FSSRNVYEVDQPSDDDRMRYLHALFESLLSFQMEESRSKSKDQKSSVDLPKAPKEVDGPKLSELFAFAEA    875
Zea_mays_NP_001105102                 FSSRNVYQVDQPSFDDRLRYFSILFESLLSFQMEESRNKSKKQKSAIDLPKAPKEVEGPKVSELFAFAEA    876
Physcomitrella_patens_XP_001760774    FG.HNCYEVKLPSTEARSKFFSQLVDAAVSVPRRRAVSRNKQKEVLPELPKAPKVVKGPSEAELREFAEM    817

Arabidopsis_thaliana_BRAT1            EQHALRRLRMCLRDVCNRILYDKRFSAFHFPVTDEIAPNYRSIIQIPMDTATLLQRVDTGQYLTCTPFLQ    960
Glycine_max_XP_003541174              EQHALRRLRMCLRDVCNRILYDKRFNAFHYPVTDEIAPNYRSIIQNPMDMATILQHVDNGHYITSAAFLQ    962
Oryza sativa Os09g0515100             EQHAVRRMRMCLRDICNRILYNKRFNVFHFPVSEEEVPDYRSVVHNPMDMATVLQQVDSGQYLTFASFMK    945
Zea_mays_NP_001105102                 EQHAVRRMRMCLRDICNRILYNKRFNVFHFPVLEDEVPDYRSIIHKPMDMATVLQRVDSGQYLTFAAFIK    946
Physcomitrella_patens_XP_001760774    EENHLRRLRMCFRDICNRLLCERRFSIFHYPVMDDEAPDYRNIVHNPMDVATLLQRVDSGCYLSKKSFLA    887

Arabidopsis_thaliana_BRAT1            DVDLIVRNAKAYNGDDYAGARIVSFAYELRDVVHGMLSQMDPALLTYCDKIAAEGGPSLIPDDLSGSILG   1030
Glycine max XP 003541174              DINLIVSNAKAYNGEDYNGARIVSFACELRIAVHGMLSQMDPALVAYCDKIASQGGPVQLSDELGDSTFP   1032
Oryza_sativa_Os09g0515100             DIDLIVSNAKTYNGSDYNGSRIVSFACELRDVVQGMLSQMDPSLVSFCDKIAEQGGPLQVTDDGDSSILQ   1015
Zea_mays_NP_001105102                 DIDLIVSNAKTYNGDDYNGSRIVSFACELRDVVQGMLSQMDPSLVSFCDKIASQGGPLQAVDDEDRAILQ   1016
Physcomitrella_patens_XP_001760774    DVDLIFANAQEYNGDDYQGGRIVSFACALRIAVLGMVSTIDPALVKICDEIAARGGPPRLPGVPSGVLVP    957

Arabidopsis thaliana BRAT1            IAPVVQMGTVTRTSARLRNVQPEVNLDRDYEGLKKPKKTTDAVSID.....SAADKSQN.QDSGQEMPSP   1094
Glycine_max_XP_003541174              ATPVVQLGQSTRMSARLRHVQPEVNMDQSYEVLKRTKK...IAEVH.....AAEEKSQ..QDS...VPS.   1088
Oryza_sativa_Os09g0515100             AAPVAQLVSGTRMSARLRNVQPEVNLSRSYEALKRQKKSTETEQGMVKESTTRDDKSLGDVDLSKPISPE   1085
Zea mays NP 001105102                 AAPVAQLVSGTRISARLRNVQPEVNLSQSYEVLRRQKKSAENEQ.....SMTRDEKSPEDVDLSKPTIAE   1081
Physcomitrella_patens_XP_001760774    TVAPP....AVRASARLRGFQPEVIALKNSDGGVRRTR................................    991

Arabidopsis thaliana BRAT1            IAANPQSAAPSPTDGDREDQSEPPSKEASAEDMSGDSCKGPAAKSDKEISSRTESVKGVEMERTDNYSIP   1164
Glycine_max_XP_003541174              KSSLEQCANDTNSERLEHVSIEGDLHGTFTNNIADGNSPDDVTVLDGEFLGEVESVKQLEVKRSENYSIP   1158
Oryza_sativa_Os09g0515100             EAPKEPDSNGVLKETDNPPTELPELPELNPEPMVTDNGENAAMPASDDIPEQLEVVKRREMELTTGYGVP   1155
Zea mays NP 001105102                 EAAKEPESNGTTKEANDSFAKEPEVSTS.PEPMESDNGKIAAATG.DDLLEQLEALKQREMELIASYGVP   1149
Physcomitrella_patens_XP_001760774    .....RSNEGLPFGKMRVGYILHKLC                                               1012

Arabidopsis_thaliana_BRAT1            QMERLYTRIMKGVLETLDKGLRDDDNNPKHSILRFLSEEAQHCAN                            1209
Glycine_max_XP_003541174              QLERLYTRIMKGVFETKNKGVSGD...LKSSVLKFLLNEVEDIAN                            1200
Oryza sativa Os09g0515100             QLERLCTRVMKGMIELSGKESNED...HRRLVVRYLLTEVENSDN                            1197
Zea_mays_NP_001105102                 QLERLYSKIMKGAIELTSKESNED...HRRLVVRYLWTEVENSNN                            1191
Physcomitrella_patens_XP_001760774
```

Supplementary Fig. 13. Alignment of BRAT1 and its conserved homologs in plants.

Supplementary Figure 14

```
Arabidopsis_thaliana_BRAT1             MHPKRSSQGDGSVTKPVRTSDRLRRRPKLHGRSYLYYSSPNMLHNRKRNTKTRTAASQIAKMLHK       65
Homo sapiens ATAD2B                   MVNTRKSSLRLLGSKSPGPGPGP..GAGAEPG...ATGGSSHFISSRTRSSKTPAASCPAAKAGGS       61
Mus_musculus_XP_006515178             MVNTRKSSLRLLGSKSPGPGPGPGPGAGAEPG...ATGGSSHFISSRTRSSKTPAASCPAAKAGGS       63
Caenorhabditis_elegans_LEX-1               MPRSDGFSPRKNLRRSARDHSRSYAGQCNEDFDDMYAPSSRRRSSGGVDGNGYTRSGRKI       60
Saccharomyces_cerevisiae_Yta7p      MARNLRNRRGSDVEIASNAKVGYETQIKDENGIIHTTTRSLRKINYAEIEKVFDFLEDDQVMDKDETPVD       70

Arabidopsis_thaliana_BRAT1          GNR.....PAPASNAAPIASDLRRSTRKRRISVNLEDYTDSSGAEDEDMMSPAYRTLRRRVHKNFSTSKS      130
Homo sapiens ATAD2B                GGAGVTLDEARKVEVDGSLSDSHVSPPAKRTLKQPDSVCKDKSKSRSTGQREEWNLSTGQARLTSQPGAT      131
Mus_musculus_XP_006515178           GGA...LDEARPAEVDGSLSDSHVSPPAKRTLKQPDSVCKDKSKSRSTGQREEWNIPSGQTRLTSQPGAT      130
Caenorhabditis_elegans_LEX-1        NHN....RYYEEEYHEAISSEEDERRYRTRRSSNSMTYRQQVMQAIDESKRNQKVPPAKRKRIYLSDEEE      126
Saccharomyces_cerevisiae_Yta7p      VTSDEHHNNNQKGDDEDDDVDLVSPHENARTNEELTNERNLRKRPAHDPEEDDESFHEEDVDDDEEEEA      140

Arabidopsis thaliana BRAT1          RKDMLAELAPRREGLRPRRSTTIANKRLK..TESGADQDTSEEKDGQDETENGNELDIADDGENEVEAED      198
Homo_sapiens_ATAD2B                 LPNGHSGLSLRSHPLRGEKKGDGDLSCIN..GDMEVRKSCRSRKNRFESVNQSLLFDQLVNSTAEAVLQE      199
Mus_musculus_XP_006515178           LPNGHSSLSLRSHPLRGEKKGDGDLSCIN..GDIEVRKSCRSRKNRFESVNQSLLFDQLVNSTAEAVLQE      198
Caenorhabditis elegans LEX-1        EDFAEAAHVENTVPERATRRSTRRRSSMH..EELGVSEQEESPVRRTRPAAKRLGSEQPEENIAADDPLP      194
Saccharomyces_cerevisiae_Yta7p      DEFEDEYLDEDSKDNNRRRPAADRKFVVPDPDDDEEYDEDDEEGDRISHSASSKRLKPANSRRTRSSRHP      210

Arabidopsis_thaliana_BRAT1          EGNGEDEGDGEDEGEEDGDDDEEGDEEQEGRKRYDLRNPAEVRRMPTGEINKQQQPRSPRRV........      260
Homo_sapiens_ATAD2B                 MDNINIRRNRRSGEVERLRMWTDTEFENMDMYSRVKRRRKSLRRNSYGIQNHHEVSTEGEEEARTSKLLP      269
Mus_musculus_XP_006515178           MDNINIRRNRRSGEVERLRMWTDTEFENMDMYSRVKRRRKSLRRNSYGIQNHHEVSTEGEEE........      260
Caenorhabditis_elegans_LEX-1        MEGGGEIVLPIAEIDGMAEQENEDLIEKIGREEEEEGAEEDEQSGEKDPEEEEDDSSNAESSEE......      258
Saccharomyces_cerevisiae_Yta7p      ETPPPVRPALRSRTRHSRTSNEENDDENDNSRNEALTIADEIRELQEDSPIREKRFLRERTKPVN.....      275

Arabidopsis_thaliana_BRAT1          ........LHQGMGTRVGRDGRRGGSRPHKRHRFTRTDDSDDSLLVDELDQGP.......AIPWARGGNR      315
Homo_sapiens_ATAD2B                 LEKISIESQEEDGDIEVEEAEGEENDRPYNLRQRKTVDRYQAPPIVPAHQKKRENTLFDIHRSPARRSHI      339
Mus_musculus_XP_006515178           ......ESQEEDGDIEVEEAEGEENDRPYNLRQRKTVDRYQAPPIVPAHQKKRENTLFDIHRSPARRSHI      324
Caenorhabditis elegans LEX-1        ......STAPRQYSLRRRQPVVQPNASEARENRRARLEHHRVANQNRHHRNRNG......SRRRRSDSDS      316
Saccharomyces_cerevisiae_Yta7p      ......YKLPPPLTASNAEEFIDKNNNNALSFHNPSPARRGRGGWNASQNSGPTRRLFPTGGPFGGNDVTT      339

Arabidopsis_thaliana_BRAT1          SGAPWLFGGLDTYGSSSLGLNVGASGWGHQSD........GIAALTSGVQTAGPSS.KGGADIQPLQINE      376
Homo_sapiens_ATAD2B                 RRKKHAIHSSDTTSSDEERFERRKSKSMAPARNRCLPMNFRAEDIASGILRERVKVGASLADVDPMNIDK      409
Mus musculus XP 006515178           RRKKHAIHSSDTTSSDEERFERRKSKSMAPARNRCLPMNFRAEDIASGILRERVKVGASLADVDPMNIDK      394
Caenorhabditis_elegans_LEX-1        DSDDMVLPRPDKRQSRPHMHNRGERERGRFMPINMTEKELQSAQHILMDRMRKTIAGQGASDIDPMSVDS      386
Saccharomyces_cerevisiae_Yta7p      IFGKNTNFYNQVPSAFSDNNNNKLILDSDSSDDEILPLGVTPKTKKENTQKKKKK.PEIADLDPLGVDM      408

Arabidopsis_thaliana_BRAT1          DINFDDIGGLSEYINDLKEMVFFPLLYPEFFASYSITPPRGVLLCGPPGTGKTLIAFALACAASKAGQKV      446
Homo sapiens ATAD2B                 SVRFDSIGGLSHHIHALKEMVVFPLLYPEIFEKFKIQPPRGCLFYGPPGTGKTLVAFALANECSQGDKKV      479
Mus musculus XP 006515178           SVRFDSIGGLSHHIHALKEMVVFPLLYPEIFEKFKIQPPRGCLFYGPPGTGKTLVAFALANECSQGDKKV      464
Caenorhabditis_elegans_LEX-1        SVGFDQVGGIGHHIQSLKEVVLFPMLYPEVFEKFRINPPKGVVFYGPPGTGKTLVAFALANECRRGANKV      456
Saccharomyces_cerevisiae_Yta7p      NVNFDDIGGLDNYIDQLKEMVALPLLYPELYQNFNITPPRGVLFHGPPGTGKTLMARALAASCSSDERKI      478

Arabidopsis_thaliana_BRAT1          SFYMRKGADVLSKWVGEAERQLKLLFEEAQRNQPSIIFFDEIDGIAPVRSSKQEQIHNSIVSTLLALMDG      516
Homo sapiens ATAD2B                 AFFMRKGADCLSKWVGESERQLRLLFDQAYLMRPSIIFFDEIDGIAPVRSSRQDQIHSSIVSTLLALMDG      549
Mus_musculus_XP_006515178           AFFMRKGADCLSKWVGESERQLRLLFDQAYLMRPSIIFFDEIDGIAPVRSSRQDQIHSSIVSTLLALMDG      534
Caenorhabditis_elegans_LEX-1        AFFMRKGADCLSKWVGESERQLRLLFDQAYAMRPSIIFFDEIDGIAPVRSSKQDQIHASIVSTLLALMDG      526
Saccharomyces_cerevisiae_Yta7p      TFFMRKGADILSKWVGEAERQLRLLFEEAKKHQPSIIFFDEIDGIAPVRSSKQEQIHASIVSTLLALMDG      548

Arabidopsis_thaliana_BRAT1          LDSRGQVVLIGATNRVDAIDGALRRPGRFDREFNFSLPGCEARAEILDIHTRKWK.HPPTRELKEEIAAT      585
Homo sapiens ATAD2B                 LDNRGEIVVIGATNRLDSIDPALRRPGRFDREFLFNLPDQKARKHILQIHTRDWN.PKLSDAFLGELAEK      618
Mus_musculus_XP_006515178           LDNRGEIVVIGATNRLDSIDPALRRPGRFDREFLFNLPDQRARKHILQIHTRDWN.PKLSDAFLGELAEK      603
Caenorhabditis_elegans_LEX-1        LDGRGEVVVIGATNRLDTLDPALRRPGRFDRELRFSLPDLNARRQILDIHTSKWEENKPIPETLDAIAER      596
Saccharomyces_cerevisiae_Yta7p      MDNRGQVIVIGATNRPDAVDPALRRPGRFDREFYFPLPDVKARFKILQIQTRKWS.SPLSTNFIDKLAFL      617

Arabidopsis thaliana BRAT1          CVGYCGADLKALCTEAAIRAFREKYPQVYTSDDKYAIDVGLVNVEKSHFVEAMSAITPAAHRGSVVQSRP      655
Homo_sapiens_ATAD2B                 CVGYCGADIKALCTEAALIALRRRYPQIYASSHKLQLDVSSIVLSAQDFYHAMQNIVPASQRAVMSSGHA      688
Mus_musculus_XP_006515178           CVGYCGADIKALCTEAALTALRRRYPQIYASSHKLQLDVTSIVLSAHDFYHAMQNIVPASQRAVMSSGQA      673
Caenorhabditis_elegans_LEX-1        TSGYCGADLKFLCTEAVLIGLRSRYPHIYMCSERLKLDVATIKITSEHFGHAMRRITPASRRDLTIPSRP      666
Saccharomyces_cerevisiae_Yta7p      TKGYGGADLRSLCTEAALISIQRSFPQIYRSNDKLLVDPSKIKVKVSDFMLALKKIVPSSARSTGSSPQP      687

Arabidopsis_thaliana_BRAT1          LSPVVLPQLHRHLLESMSLISDIFPSSATSSELTKLSILTFGS...........................      698
Homo_sapiens_ATAD2B                 LSPIIRPLLERSFNNIIAVLQKVFPHAEISQSDKKEDIETLILEDSEDENALSIFETNCHSGSPKKQSSS      758
Mus_musculus_XP_006515178           LSPIIRPLLERSFNNIIAVLQKIFPHAETSQSDKKEDVGSLILDDSEEENALSIFEMSCHSGSPKKSLPA      743
Caenorhabditis_elegans_LEX-1        IDERTSILLGDTVSNLISLRIPQGYRCVENAMATASSELEQVVRALE.......................      713
Saccharomyces_cerevisiae_Yta7p      LPELIKPLLADQLNNLKNKLDYMLNIKDTTFQRNTSLLQNFIDYEEYSGE..........EEEHDKYGGN      747
```

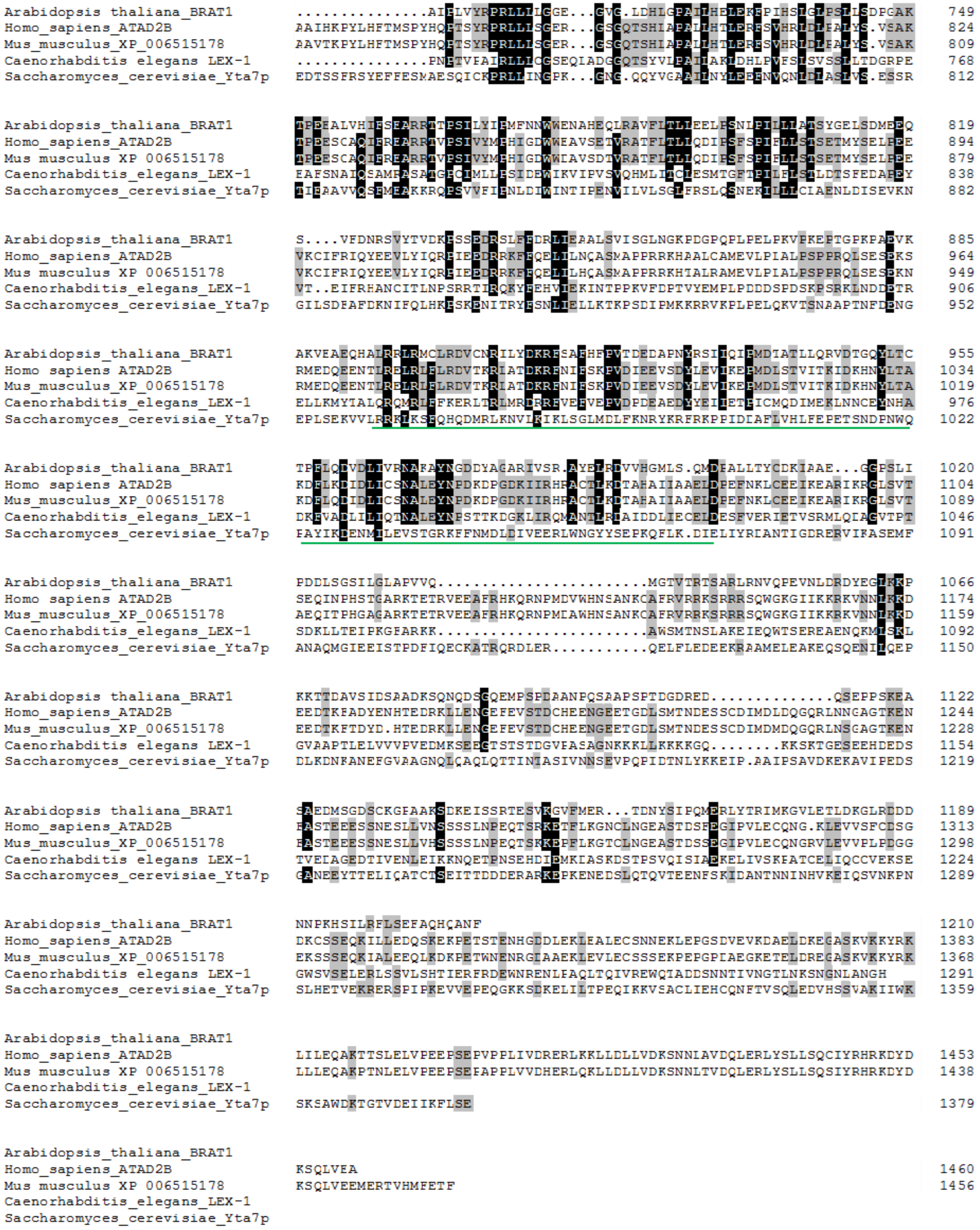

Supplementary Fig. 14. Alignment of BRAT1 and its homologs in eukaryotic organisms. The ATPase domain and bromodomain of BRAT1 are represented by red and green lines, respectively.

Supplementary Figure 15

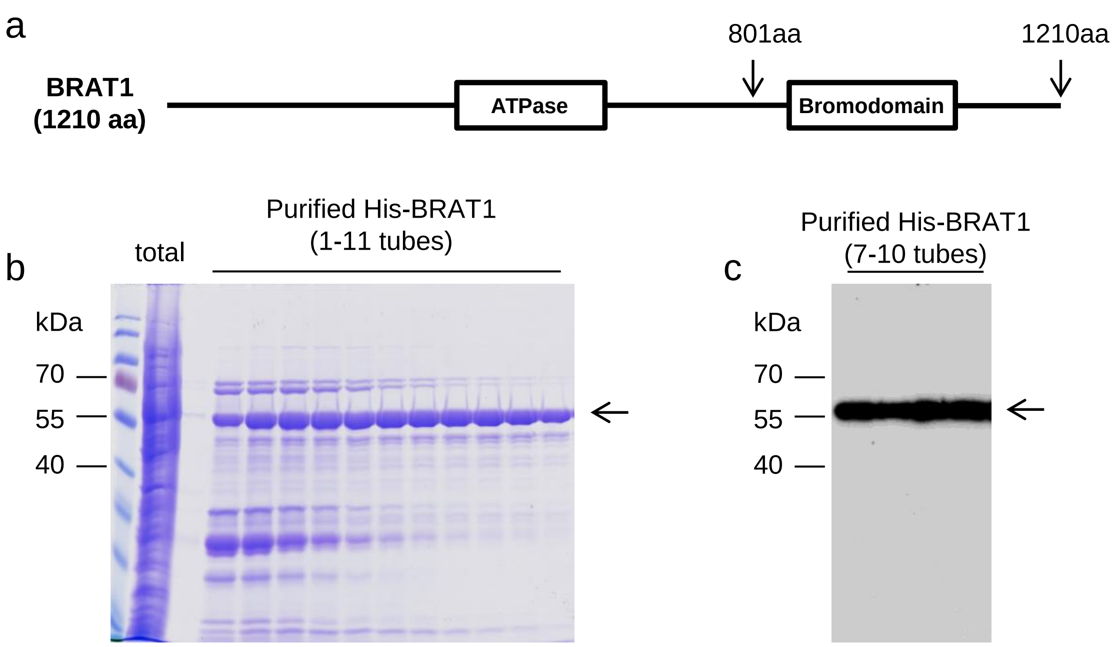

Supplementary Fig. 15. The bromodomain of BRAT1 fused with His tag was purified. (**a**) Diagram of the full-length BRAT1 protein. (**b**) The purified His-BRAT1 (801–1210 aa) was subjected to SDS-PAGE and stained with Coomassie Blue. (**c**) The purified His-BRAT1 (801–1210 aa) was analyzed by Western blotting with His antibody. The arrow indicates the band of His-BRAT1 (801–1210 aa).

Supplementary Figure 16

Location	Name	Mod 1	Mod 2	Mod 3	Mod 4	Intensity
L12	H4 1-19	unmod				0.290816
L16	H4 1-19	K5ac				0.154872
L17	H4 1-19	K8ac				0.191738
L18	H4 1-19	K12ac				0.138743
L19	H4 1-19	K16ac				0.107472
M3	H4 1-19	K5ac	K8ac			0.944536
M4	H4 1-19	K8ac	K12ac			1
M5	H4 1-19	K8ac	K16ac			0.982225
M9	H4 1-19	R3me2s	K5ac	K8ac		0.733048
M10	H4 1-19	R3me2a	K5ac	K8ac		0.873272
M11	H4 1-19	K5ac	K8ac	K12ac		0.837393
M12	H4 1-19	K8ac	K12ac	K16ac		0.9842
M13	H4 1-19	S1P	R3me2s	K5ac	K8ac	0.852535
M15	H4 1-19	R3me2s	K5ac	K8ac	K12ac	1
M16	H4 1-19	R3me2a	K5ac	K8ac	K12ac	1
M17	H4 1-19	K5ac	K8ac	K12ac	K16ac	0.391047
M18	H4 11-30	unmod				0.111257
N4	H4 11-30	K20ac				0.597433
N7	H4 11-30	K12ac	K16ac			0.578506
N15	H4 11-30	K16ac	K20ac			0.793779
N16	H4 11-30	K12ac	K16ac	K20me1		0.274358
N17	H4 11-30	K12ac	K16ac	K20me2		0.058756
N18	H4 11-30	K12ac	K16ac	K20me3		0.160961
N19	H4 11-30	K12ac	K16ac	K20ac		0.238808
N23	H4 11-30	R19me2a	K20ac			0.033739
O3	H4 11-30	R19me2s	K20ac			0.953423

Supplementary Fig. 16. The spot intensity of the histone peptide array was analyzed using Active Motif Array analyze software. Gray boxes indicate the peptides on the array that were specifically bound by the bromodomain of BRAT1.

Supplementary Figure 17

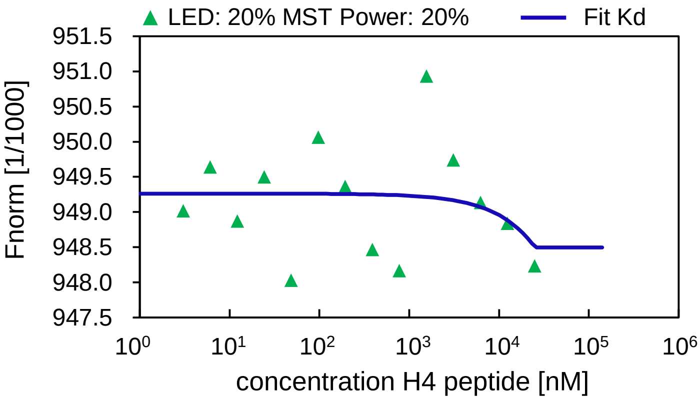

Supplementary Fig. 17. Non-acetylated H4 was used as a negative control in MST assay, and showed no binding to the bromodomain of BRAT1.

Supplementary Figure 18

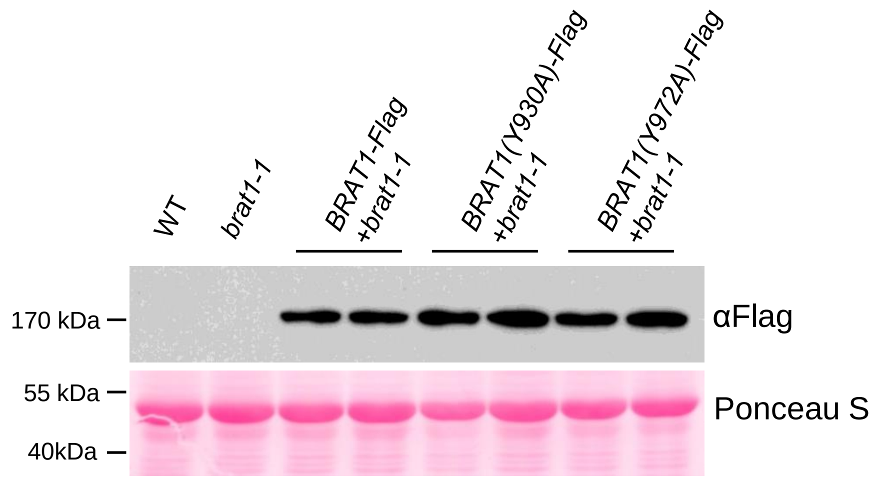

Supplementary Fig. 18. The expression levels of the wild-type and mutated *BRAT1-Flag* transgenes are similar in the *brat1-1* mutant background. The expression levels were determined by Western blot assay. Ponceau S staining of Rubisco is shown as a loading control.

Supplementary Figure 19

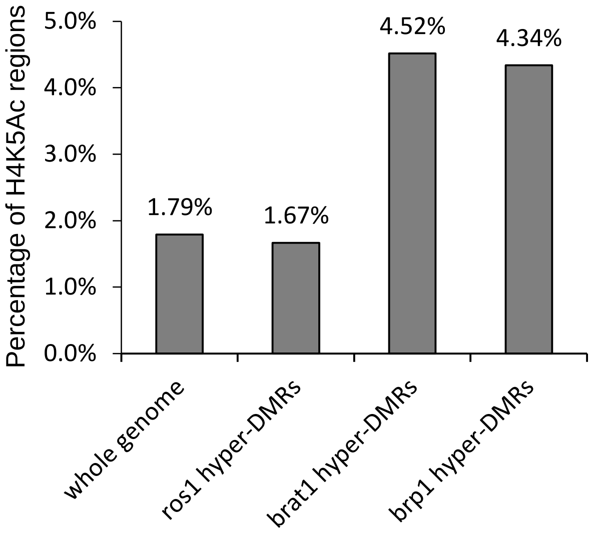

Supplementary Fig. 19. Percentage of H4K5Ac regions in the whole genome and in the hyper-DMRs in *ros1*, *brat1*, and *brp1*.

Supplementary Figure 20

Fig. 2b

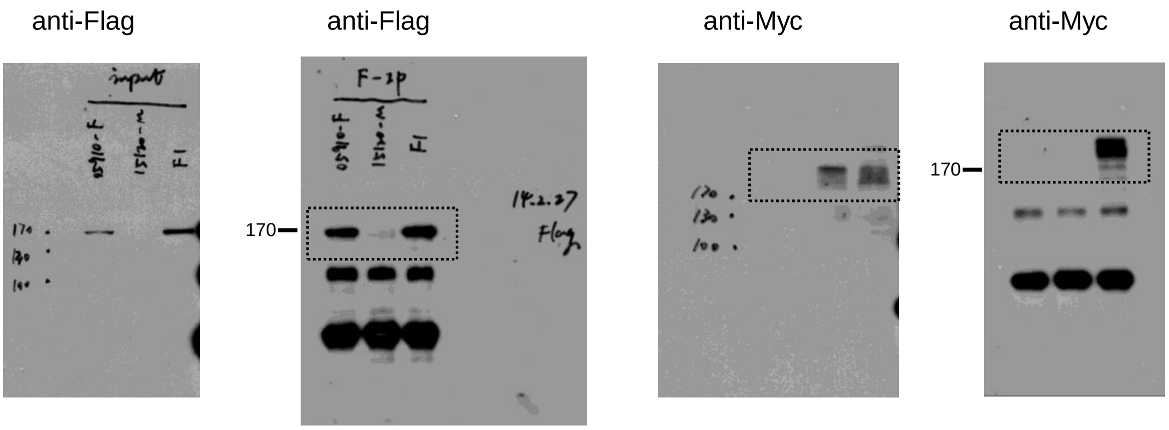

Fig. 2c Left

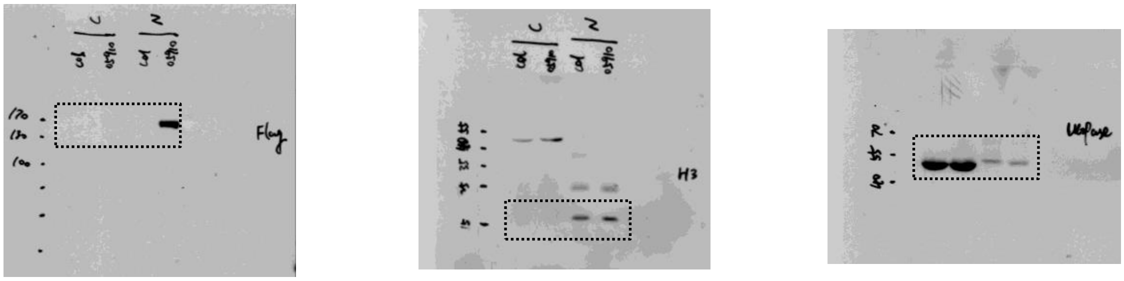

Fig. 2c Right

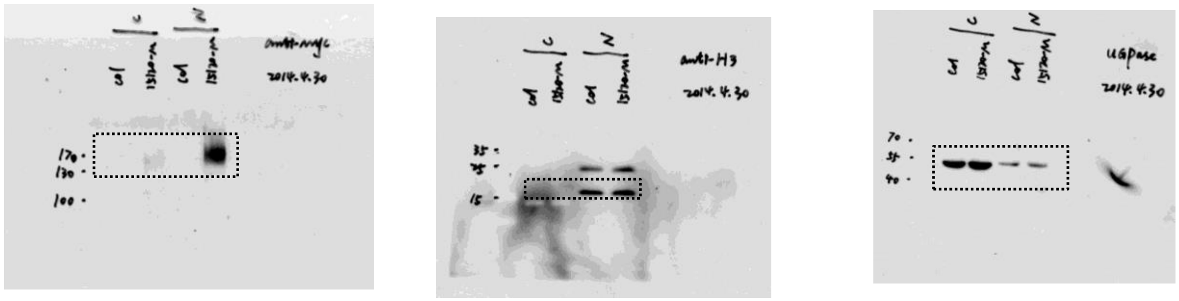

Fig. 7b

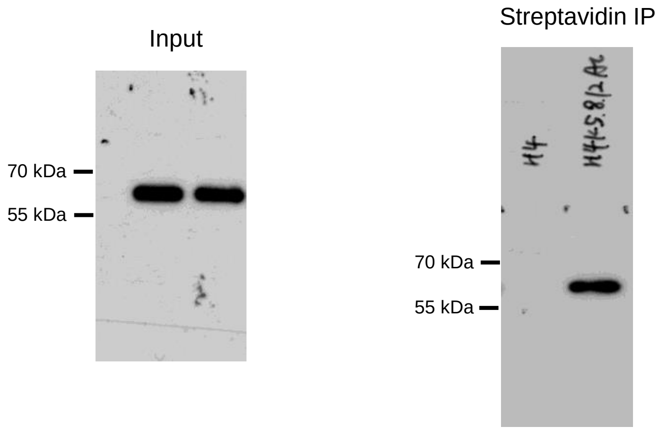

Fig. 7c

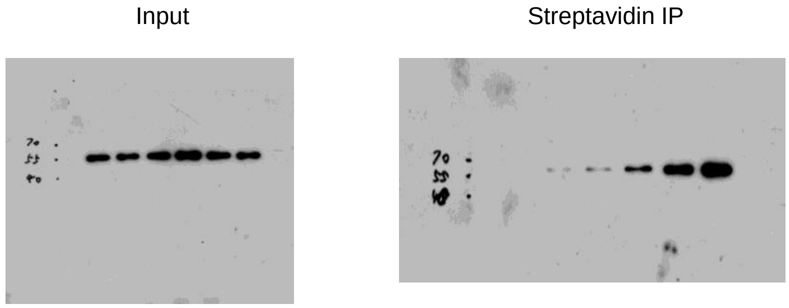

Fig. 7e

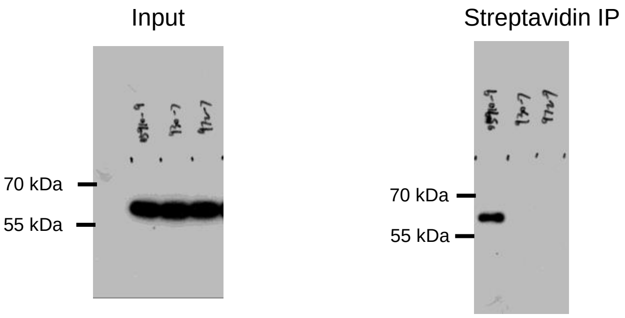

Sup. Fig. 1b

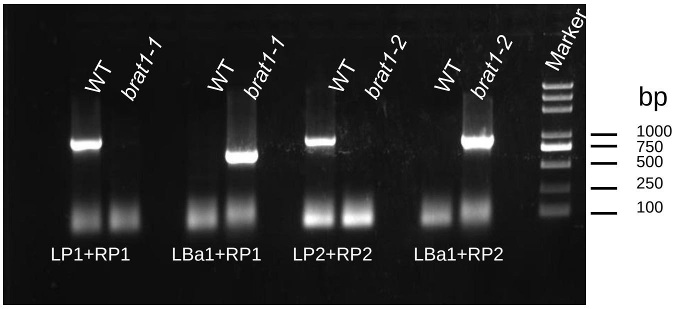

Sup. Fig. 1c

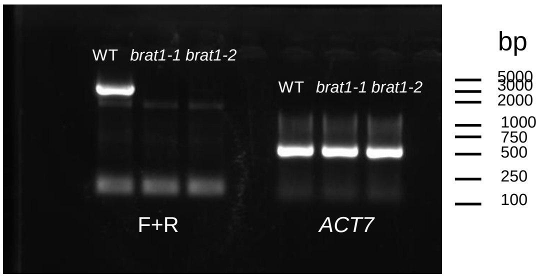

Sup. Fig. 1d

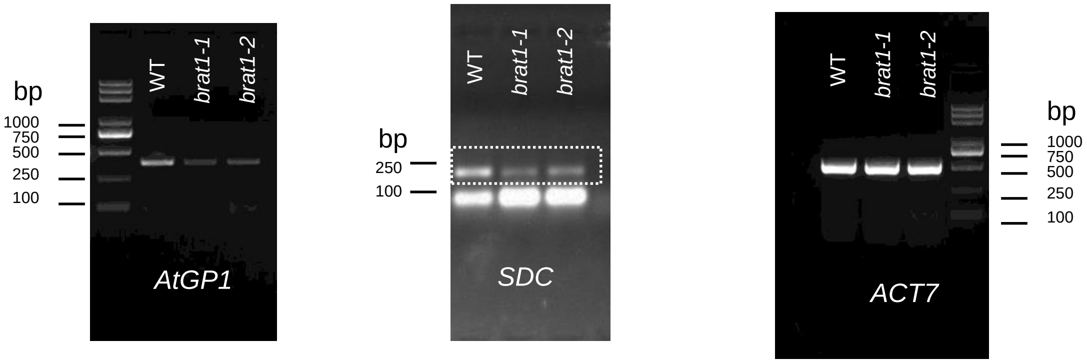

Sup. Fig. 2

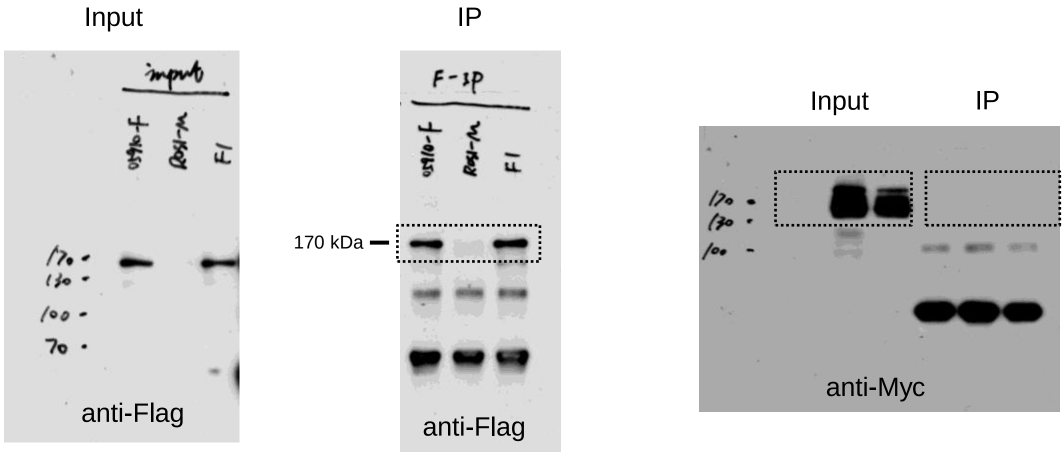

Sup. Fig. 3

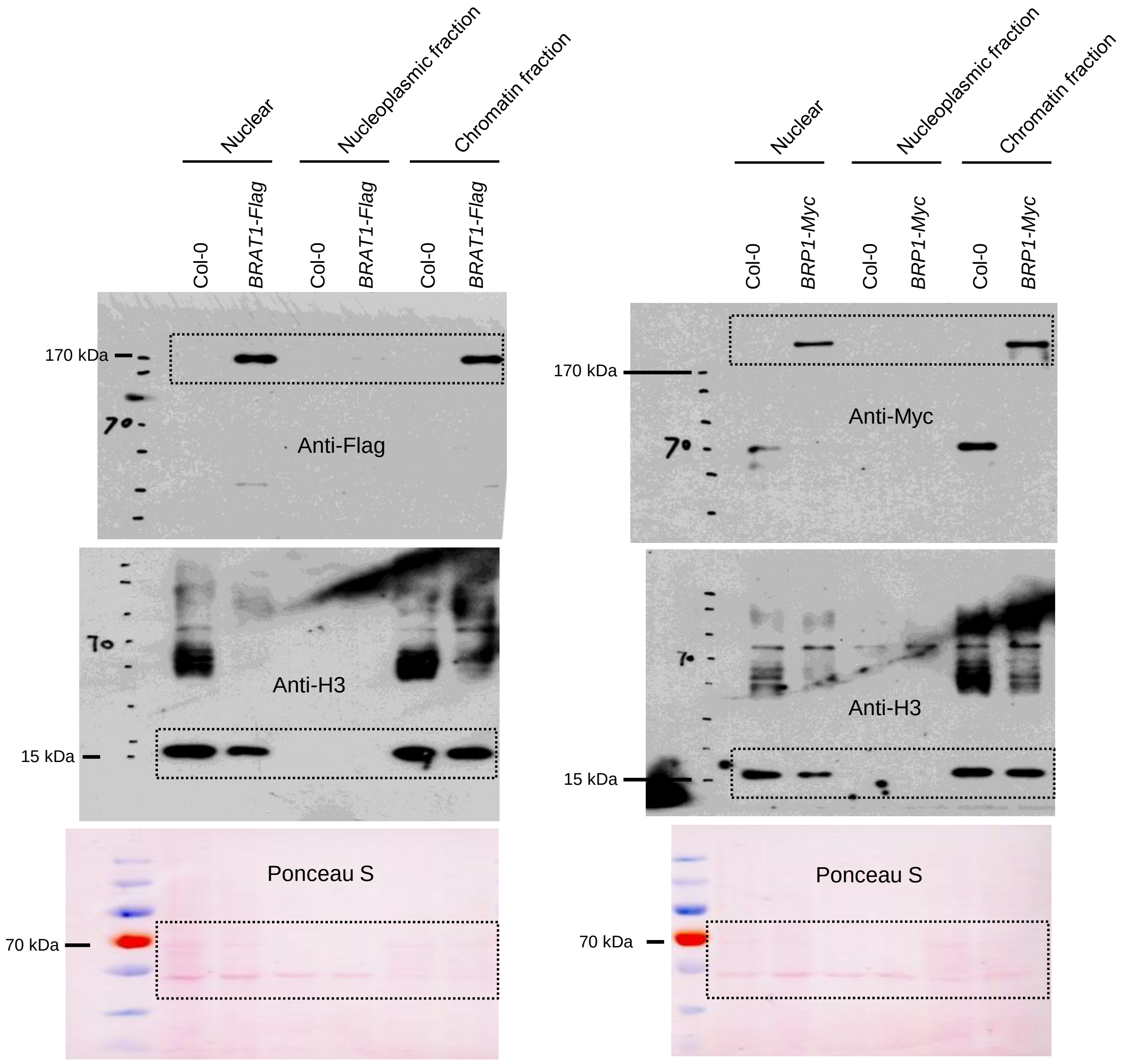
Nuclear
Nucleoplasmic fraction
Chromatin fraction
Col-0
BRAT1-Flag
170 kDa
70
Anti-Flag
Anti-H3
15 kDa
Ponceau S
70 kDa
Nuclear
Nucleoplasmic fraction
Chromatin fraction
Col-0
BRP1-Myc
170 kDa
70
Anti-Myc
Anti-H3
15 kDa
Ponceau S
70 kDa

Sup. Fig. 4c

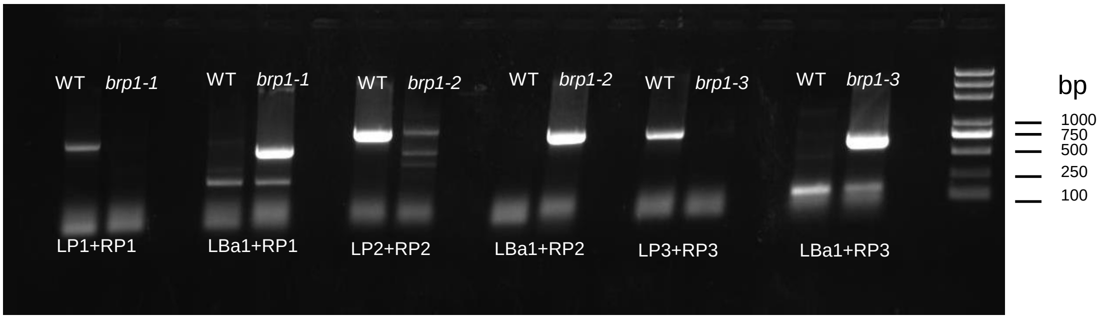

Sup. Fig. 4d

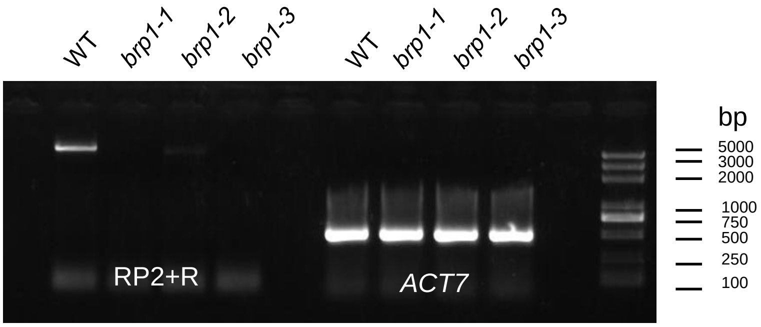

Sup. Fig. 6a

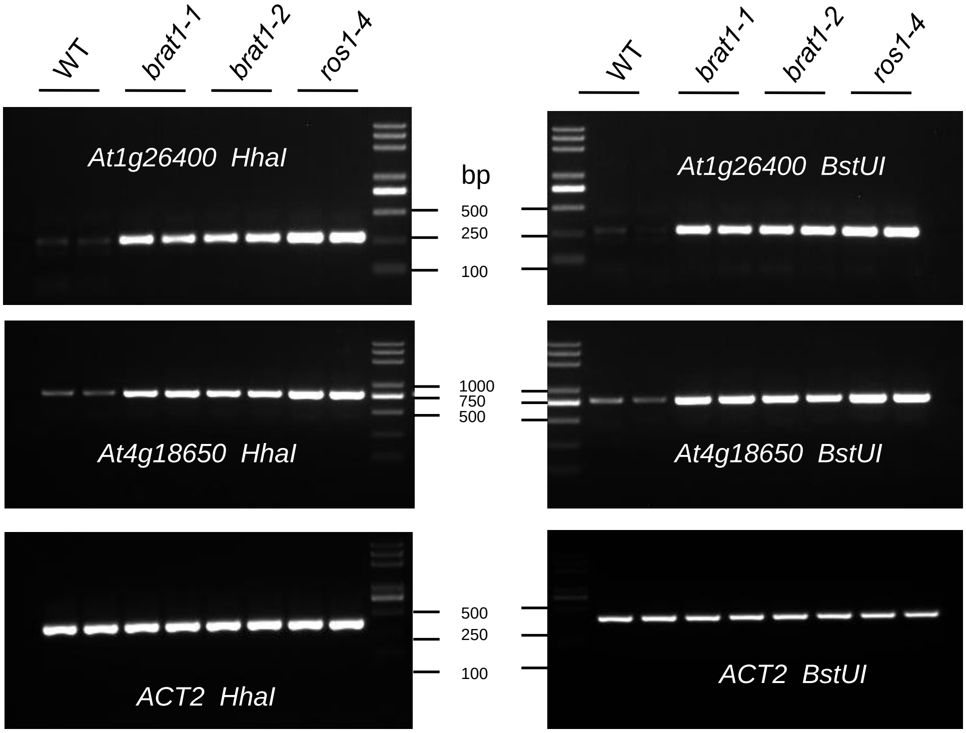

Sup. Fig. 6b

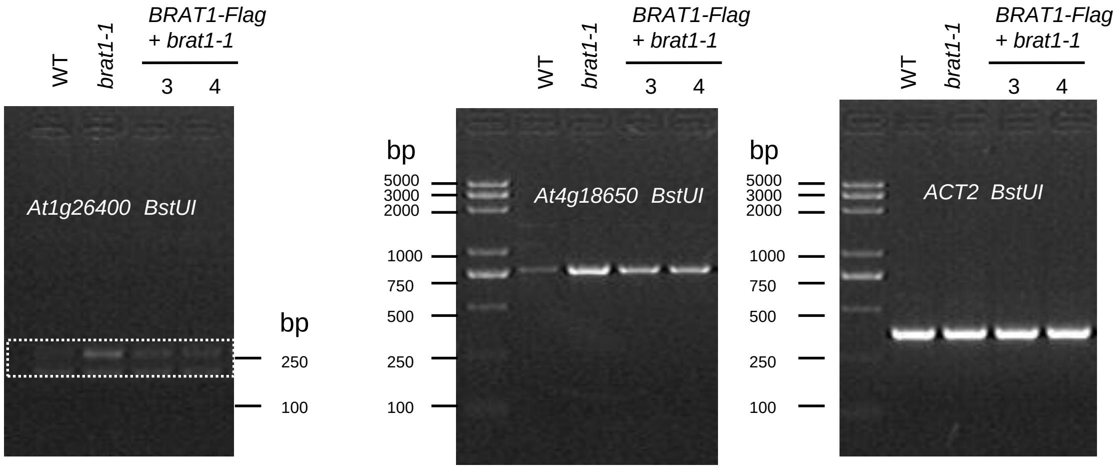

Sup. Fig. 6c

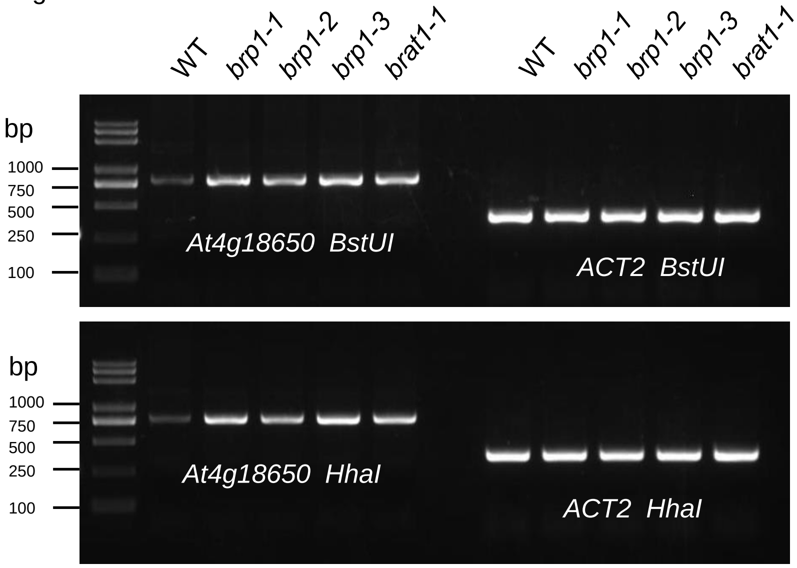

Sup. Fig. 6d

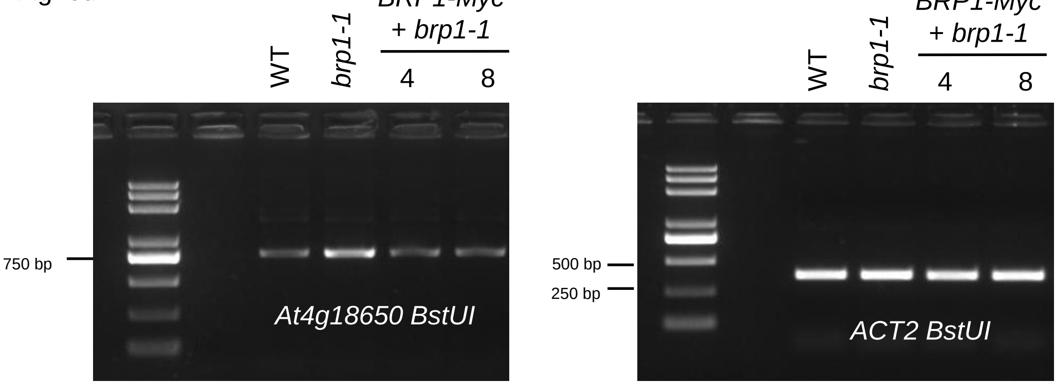

Sup. Fig. 18

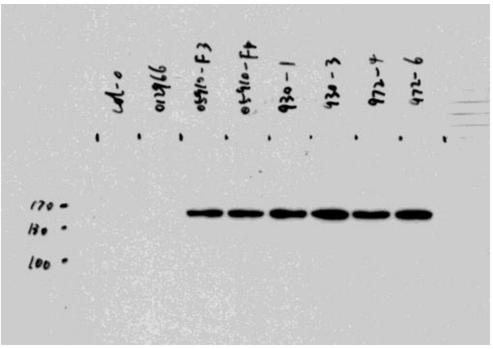

αFlag

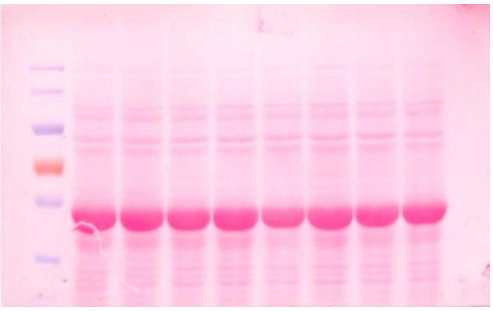

Ponceau S

Supplementary Fig. 20. Full-size images of gels and western blots shown in this study.

Supplementary Table 1. Summary of bisulfite sequencing data.

Sample	Uniquely Mapping Reads	Coverage (X)	CpG error rate	CHG error rate	CHH error rate
WT	28606152	21.25	0.31%	0.32%	0.34%
brat1-1	31236082	23.20	0.31%	0.32%	0.34%
brp1-1	48639162	36.12	0.31%	0.32%	0.34%
ros1-4	46396604	34.46	0.33%	0.34%	0.36%

Supplementary Table 2. Summary of reads from RNA deep sequencing analysis.

Sample	Total reads	Reads mapped on genome	Ratio
WT_rep1	46520424	45582182	97.98%
WT_rep2	15127064	14315271	94.63%
WT_rep3	20453402	19290154	94.31%
brat1_rep1	45408383	44487901	97.97%
brat1_rep2	19049645	17954986	94.25%
brat1_rep3	18500671	17475674	94.46%
nrpe1_rep1	41774171	40891648	97.89%
nrpe1_rep2	17540787	16504061	94.09%
nrpe1_rep3	17069612	16066757	94.12%
nrpe1xbrat1_rep1	46202262	45195593	97.82%
nrpe1xbrat1_rep2	18962020	17830415	94.03%
nrpe1xbrat1_rep3	17601679	16632842	94.50%
